# Auditory and cognitive factors underlying individual differences in aided speech-understanding among older adults

**DOI:** 10.3389/fnsys.2013.00055

**Published:** 2013-10-01

**Authors:** Larry E. Humes, Gary R. Kidd, Jennifer J. Lentz

**Affiliations:** Department of Speech and Hearing Sciences, Indiana UniversityBloomington, IN, USA

**Keywords:** presbycusis, speech recognition, amplification, psychoacoustics, aging

## Abstract

This study was designed to address individual differences in aided speech understanding among a relatively large group of older adults. The group of older adults consisted of 98 adults (50 female and 48 male) ranging in age from 60 to 86 (mean = 69.2). Hearing loss was typical for this age group and about 90% had not worn hearing aids. All subjects completed a battery of tests, including cognitive (6 measures), psychophysical (17 measures), and speech-understanding (9 measures), as well as the Speech, Spatial, and Qualities of Hearing (SSQ) self-report scale. Most of the speech-understanding measures made use of competing speech and the non-speech psychophysical measures were designed to tap phenomena thought to be relevant for the perception of speech in competing speech (e.g., stream segregation, modulation-detection interference). All measures of speech understanding were administered with spectral shaping applied to the speech stimuli to fully restore audibility through at least 4000 Hz. The measures used were demonstrated to be reliable in older adults and, when compared to a reference group of 28 young normal-hearing adults, age-group differences were observed on many of the measures. Principal-components factor analysis was applied successfully to reduce the number of independent and dependent (speech understanding) measures for a multiple-regression analysis. Doing so yielded one global cognitive-processing factor and five non-speech psychoacoustic factors (hearing loss, dichotic signal detection, multi-burst masking, stream segregation, and modulation detection) as potential predictors. To this set of six potential predictor variables were added subject age, Environmental Sound Identification (ESI), and performance on the text-recognition-threshold (TRT) task (a visual analog of interrupted speech recognition). These variables were used to successfully predict one global aided speech-understanding factor, accounting for about 60% of the variance.

## Introduction

The prevalence of hearing loss among adults over the age of 60 years has been estimated to be about 40% (e.g., Cruickshanks, 2010). A common consequence of the presence of such hearing loss is difficulty understanding speech in many everyday listening situations, but especially those situations involving backgrounds of competing speech or noise. For older adults, however, declining hearing sensitivity may not be the only factor contributing to the speech-understanding difficulties experienced. A review of the literature on factors contributing to the speech-understanding problems of older adults was provided by a working group of the Committee on Hearing and Bioacoustics and Biomechanics (CHABA) of the National Research Council (CHABA, 1988). Basically, as noted in Humes (1996), this report reviewed the evidence available at that time and evaluated this evidence relative to its support for one of three site-of-lesion hypotheses: (1) peripheral, which focused on the cochlea, and included both a simple (audibility loss) and more complex (suprathreshold processing deficits associated with cochlear pathology) version of the hypothesis; (2) central auditory, which included auditory centers of the brainstem and cortex; and (3) cognitive, which involved non-auditory areas of the cortex used in various aspects of linguistic and cognitive processing. As noted in the CHABA report, these hypotheses were not mutually exclusive and any or all of them could apply to a given study or a given individual.

Since the publication of the CHABA report, there have been numerous studies conducted in an effort to better understand the factors contributing to the speech-understanding difficulties of older adults. Early post-CHABA studies emphasized peripheral and cognitive factors (e.g., van Rooij et al., 1989; van Rooij and Plomp, 1990, 1992) or peripheral, central-auditory, and cognitive factors (Jerger et al., 1989, 1991; Humes et al., 1994). In these and similar studies through the three decades following the CHABA report, the primary focus was on the understanding of unamplified speech by older adults; that is, speech presented at conversational levels of 60–70 dB SPL and without the use of amplification to compensate for the peripheral hearing loss. Repeatedly and consistently across studies, hearing thresholds were found to be the most significant contributor to individual differences in unaided speech understanding by older adults, often accounting for 30–80% of the total variance in speech-understanding performance [see review by Humes and Dubno (2010)]. This was especially true for listening in quiet and steady-state background noise, which were the two conditions that received the most attention initially from many researchers. Often, in studies involving speech stimuli presented in competing speech or speech-like (fluctuating) backgrounds, however, cognitive factors emerged as minor, but statistically significant secondary contributors to individual differences in speech-understanding performance [see reviews by Akeroyd (2008); Houtgast and Festen (2008)].

Over the past decade or so, there has been increased interest in the factors underlying individual differences in speech-understanding performance for older adults when listening to amplified speech. Amplified speech, appropriately implemented, has the capability of overcoming the inaudibility of speech resulting from peripheral hearing loss. As was the case for unamplified speech, some researchers over the past decade chose to evaluate peripheral, central auditory, and cognitive factors as potential explanatory factors (e.g., Humes, 2002) whereas many more focused exclusively on peripheral and cognitive factors (Akeroyd, 2008; Houtgast and Festen, 2008; Humes and Dubno, 2010). For amplified speech, the relative contributions of peripheral and cognitive factors differed from that found with unamplified speech, for which hearing sensitivity was clearly the dominant factor. Rather, cognitive factors were typically found to be at least as important as hearing loss and were often the predominant factor accounting for individual differences in *aided* speech-understanding performance (Akeroyd, 2008; Houtgast and Festen, 2008; Humes and Dubno, 2010). Again, this is most apparent for speech-understanding performance measured in a background of competing speech or speech-like stimuli and may also depend upon the complexity of the target speech materials, the response task, or both. For the most part, studies of individual differences in speech understanding for amplified speech by older listeners have had relatively small numbers of older subjects (typically less than 25), making any conclusions based on the examination of individual differences tenuous at best. Humes (2002) is an exception, having tested 171 older adults, but this study made use of clinical hearing aids and full restoration of speech audibility was not achieved (Humes, 2002, 2007). Perhaps this is why Humes (2002) also saw much greater relative importance of peripheral hearing loss compared to cognition as a predictor of individual differences in the perception of amplified speech.

The present study sought to remedy some of the weakness of prior studies of individual differences in the perception of amplified speech by older adults. A relatively large sample of older adults (*N* = 98) was studied. Moreover, to ensure sufficient audibility, at least through 4000 Hz, spectral shaping of speech was applied in a laboratory setting, rather than relying on the use of clinical hearing aids. In addition, psychophysical measures of auditory processing thought to be relevant to the perception of speech in competing speech were added to a test battery of peripheral and cognitive measures administered to all subjects. Several of these auditory psychophysical measures, all making use of non-speech stimuli, were designed to tap processes related to the encoding of temporal information, such as slow envelope fluctuations and faster periodicity information that may be important for the segregation of target and competing talkers. Others were designed to assess various aspects of energetic and informational masking. Finally, a wide array of speech-understanding measures was included in this study to provide a more complete assessment of the difficulties experienced by older adults. Most of the speech-understanding measures involved competing speech-like backgrounds whereas others were included to verify restoration of performance to high levels with amplification in the absence of competition. Additional details about the measures included can be found in the next section.

The focus of this paper is on the individual differences in the understanding of amplified speech by the 98 older adults included in this study. However, we thought it was important to also test a small group (*N* = 27) of young normal-hearing adults for comparison purposes because several of the measures described below had not been used in an identical fashion with either younger or older adults previously. In addition, for those prior studies with young adults using similar paradigms the sample sizes were often small (*N* < 10). Between-group comparisons could then inform us as to whether older adults with impaired hearing performed differently relative to a young normal-hearing comparison group. Further, it has often been observed that tests believed to be reliable, based on data from young normal-hearing adults, do not prove to be reliable when evaluated in older adults (e.g., Dubno and Dirks, 1983; Christopherson and Humes, 1992; Cokely and Humes, 1992; Humes et al., 1996). Since reliability data were not available for many of the measures developed for this study, the test-retest reliability was examined in a subgroup (*N* = 31) of the 98 older adults. After methodological details have been presented, the results will be presented for the reliability analyses, followed by the age group comparisons, and then the examination of individual differences among the 98 older adults.

## Methods

### Subjects

**Figure 1:**
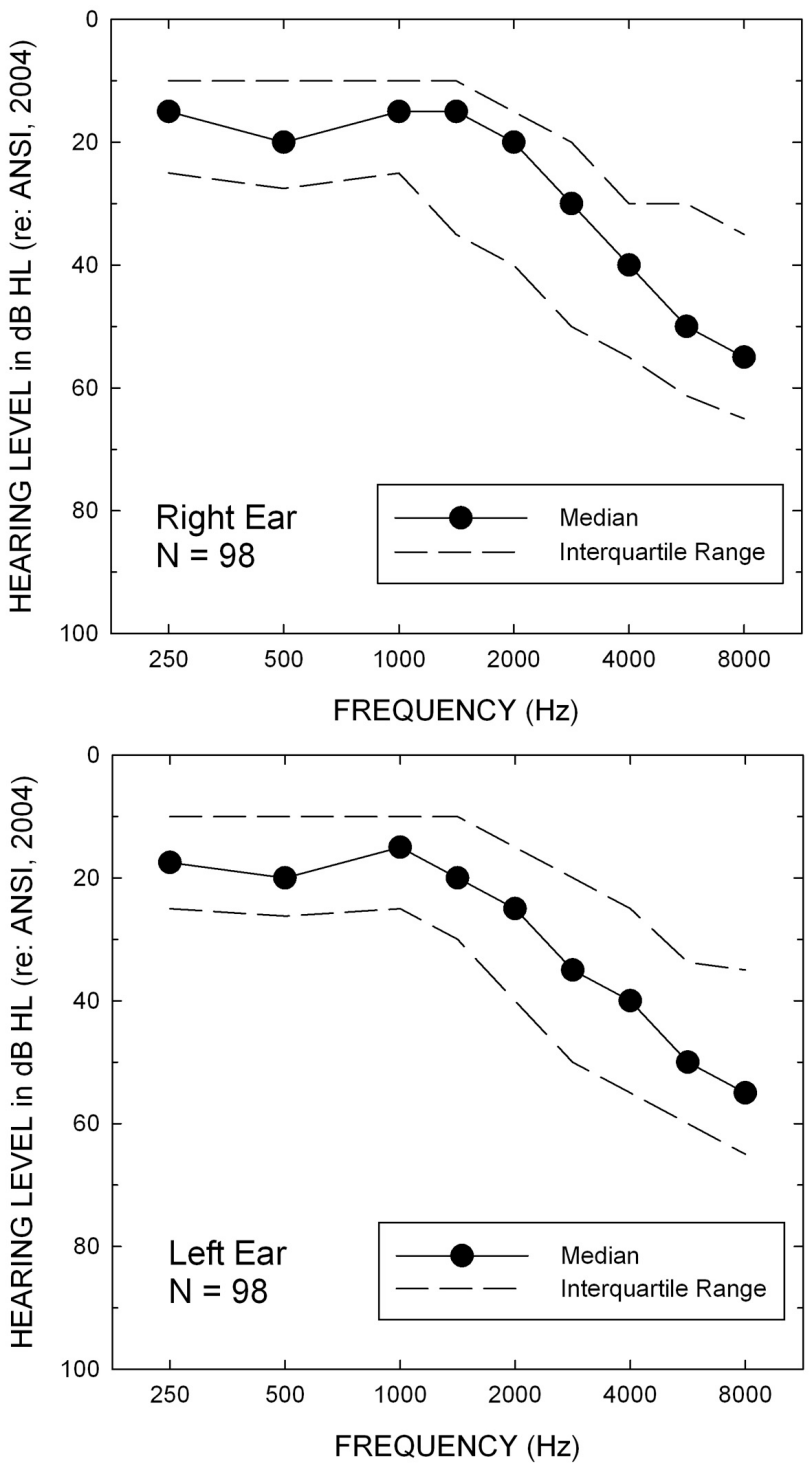
**Median hearing thresholds (filled circles) and interquartile ranges for the right (top) and left (bottom) ears of the 98 older adults in this study**.

The 98 older adults included in this study ranged in age from 60 to 86 years (*M* = 69.2 y). Fifty were female, 91 were not current hearing aids users, and 88 had never worn hearing aids. Their hearing was characterized by a bilaterally symmetrical high-frequency sensorineural hearing loss of varying degrees and the median audiograms for each ear are shown in Figure 1. Most (91 of 98) subjects had their right ears tested and the remainder had their left ear tested for all monaural measures. The left ear was tested whenever the hearing in the right ear was too severe for inclusion in this study. In addition to an inclusion criterion based on the severity of hearing loss, all subjects had no evidence of middle-ear pathology (air-bone gaps <10 dB and normal tympanograms bilaterally), no signs of dementia (Mini Mental Status Exam, MMSE, >25; Folstein et al., 1975), and had English as his or her native language. Subjects were recruited primarily via newspaper ads in the local paper and were paid for their participation.

The 27 young normal-hearing adults ranged in age from 18 to 30 years (*M* = 22.7 y) and 21 were female. These subjects had hearing thresholds ≤25 dB HL (ANSI, 2004) from 250 through 8000 Hz in both ears, no evidence of middle-ear pathology, no signs of dementia (MMSE > 25), and had English as his or her native language. Mean audiometric thresholds for every frequency and both ears were less than 10 dB HL. The test ear was always the right ear for the young subjects. Subjects were recruited primarily by flyers and university online postings. Young subjects were also paid for their participation.

### Psychophysical non-speech measures

#### Common procedures

For the auditory psychophysical measures, except for streaming, thresholds were measured for a variety of tasks using a standard/two-alternative forced-choice method, with trial-by-trial signal strengths chosen according to a 2-down, 1-up adaptive staircase procedure estimating the 70.7% correct point on the psychometric function (Levitt, 1971). In this procedure, a comparison stimulus was always presented in the first interval, and the second and third intervals contained a target and a comparison stimulus presented in random order with equal likelihood. Listeners were provided a visual marker for each observation interval and indicated which interval contained the target (altered) stimulus by responding to the appropriate area displayed on a touch screen monitor. Correct-answer feedback was provided to the listener following each trial. The staircase continued until a total of 7 reversals of the direction of the track were obtained. The mean of the signal strengths (geometric mean for harmonic mistuning) at the last 6 reversal points was taken as threshold. Unless noted otherwise, all psychophysical measures were repeated five times with those five replicates averaged to form a single threshold estimate. Analyses of performance across the five replicates failed to reveal consistent significant trends over time and the five replicates were averaged for all subjects and tasks as a result. As will be demonstrated below, most of these measures were found to be reliable in the older adults, the age group most likely to show changes in performance over time. It is acknowledged, however that the average of five relatively brief estimates of performance on a given task, typically representing a total of 200–250 trials, is not likely to be representative of asymptotic levels of performance, for either age group, that could be obtained with a much larger number of trials. All testing was conducted in a sound-attenuating room.

All stimuli were digitally generated and played through one or two channels of a 24-bit digital-to-analog converter (DAC; TDT System III RP2.1) at a sampling rate of 4096 × 10^−5^ s (about 24,414 Hz). The output was fed into Etymotic Research ER-3A insert earphones. Stimuli were presented monaurally in most of the tests, except for informational masking and masking-level-difference experiments which included presentation to both ears. Each measure is described in more detail below.

#### Informational masking

These measures were developed from consideration of the work of Kidd et al. (1994). The task was to detect a series of fixed-frequency tone bursts (i.e., a signal train) embedded in a masker having similar temporal characteristics to the signal, but randomly selected frequency characteristics. Signal and masker stimuli were eight 60-ms tone bursts with 10-ms rise/fall times presented sequentially for a total stimulus duration of 480 ms. The interval between successive tone bursts was 0 ms as in Kidd et al. (1994). The signal contained pure-tone bursts at 500 or 1000 Hz, with a fixed frequency and phase across the eight bursts. In the masker stimulus, the bursts were 6-tone complexes with randomly selected frequencies. The frequencies were chosen from frequencies ranging from 2 octaves below to 2 octaves above the signal frequency, with the restriction that no component fall within ±12% of the signal frequency. In this task, the target stimulus was generated by adding the signal and masker stimuli, whereas the comparison stimulus was the masker alone stimulus. Two different masker types were tested: burst-same and burst-different. In the burst-same condition, the frequencies and phases of the masker bursts were held fixed across the eight bursts, and a new masker was generated for each interval. In the burst-different condition, the frequencies and phases were randomly selected across bursts and across intervals.

The masker level was fixed at 80 dB SPL per component. Stimuli were presented diotically to both ears, with a 700 ms inter-stimulus interval. Threshold is expressed as the signal level in dB SPL needed for detection. At the beginning of every track, the signal strength was set to 95 dB SPL. The initial step size of the tracking procedure was 5 dB, and after three reversals the step size was reduced to 2.5 dB. The signal level could never exceed 105 dB SPL, and conditions were ordered such that the burst-different conditions were always presented first and the signal frequency (500 or 1000 Hz) was selected at random for each adaptive run. A single threshold was measured in each condition prior to obtaining repeat thresholds in the various conditions.

#### Modulation detection

This task measured the just-detectable modulation depth of sinusoidal amplitude modulation imposed on broadband noise (e.g., Viemeister, 1979; Bacon and Viemeister, 1985). The carrier, broadband noise, was generated using a Gaussian random generator. When modulation was present, the modulated stimulus was generated by multiplying the carrier by a raised cosine with a modulation rate of *f*_*m*_ (5, 20, or 60 Hz), an amplitude (modulation depth) of *m* (where *m* = modulator amplitude/carrier amplitude), and a random starting phase. The comparison stimuli were unmodulated carriers, and the target stimulus was the modulated carrier. A new random noise was chosen for each interval. Listeners detected which stimulus contained the modulation, and the just detectable modulation is expressed as 20log*m*.

Stimuli were calibrated such that the modulated and unmodulated stimuli were 80 dB SPL. Stimuli were 400 ms in duration with 40-ms rise/fall times, separated by a 600-ms interstimulus interval. The initial modulation depth was 0 dB (fully modulated; *m* = 1). The step size was 5 dB, decreasing to 2 dB after 3 reversals. Thresholds were tested using a random ordering of the modulation rates, with the caveat that all three modulation rates were tested before replicates were obtained.

#### Modulation detection interference (MDI)

This task was similar to modulation detection, but the carrier was a tone rather than noise, and the just-detectable modulation depth was measured in the presence of a high-frequency tone, which when modulated, interferes with modulation detection of the low-frequency tone (e.g., Yost et al., 1989). The standard stimulus was an unmodulated 400-Hz tone added to a 1974-Hz tone (with random phases), which could be either unmodulated or modulated. The comparison (target) stimulus was generated by modulating the 400-Hz carrier with a sinusoid of modulation depth, *m*, a modulation rate of 5, 10, or 20 Hz, and a random starting phase. In the no-interference conditions, the 1974-Hz tone was not modulated. In the interference conditions, the 1974-Hz tone was 100% amplitude modulated at a rate equal to the modulation rate imposed on the 400-Hz tone and the starting phase was random. Note that both target and comparison stimuli contain 400-Hz and 1974-Hz components. Listeners detected which interval contained the modulated 400-Hz tone, and thresholds are expressed as 20log*m*.

The modulated and unmodulated tones were calibrated such that their average overall level was 80 dB SPL. Each stimulus was 400 ms with 40-ms rise/fall times, and the interstimulus interval was 600 ms. The initial signal strength was 0 dB (fully modulated; *m* = 1), and the adaptive tracking procedure used a step size of 5 dB which was decreased to 2 dB after 3 reversals. Thresholds were measured by randomizing the modulation rate and the interference types without blocking. Each rate and interference combination was tested before replicates were obtained.

#### Masking level difference (MLD)

In this task, listeners detected a tone added to Gaussian noise. The comparison stimuli were broadband noises presented diotically, with the same noise presented to each ear. The target stimulus was generated by adding a tone of either 250 or 500 Hz with random phase to a different noise. As with the comparison stimulus, the noise in the target stimulus was presented diotically. In the NoSo conditions, the tone was presented diotically, in phase across the ears. In the NoSπ condition, the tone was presented dichotically, 180° out of phase across the ears (e.g., Hirsh, 1948). Listeners were asked to detect the tone added to the band of noise.

The noise was presented at an overall level of 80 dB SPL. The stimuli were 250 ms in duration with 40-ms rise/fall times, and the interstimulus interval was 500 ms. In the adaptive track, the initial signal level was 80 dB SPL, and the step size was 5 dB, decreasing to 2 dB after 3 reversals. The maximum permissible signal level was 105 dB SPL. Thresholds were obtained using a randomized block design in which the signal frequency was selected at random, and NoSo and NoSπ conditions were tested before moving on to the next frequency. A single threshold was obtained for each frequency/condition combination prior to obtaining repeat estimates.

#### Anisochrony

In this task, the listeners were asked to detect a lengthened inter-onset interval (IOI) embedded in an otherwise isochronous tone sequence. Comparison tone sequences consisted of eight 50-ms tones with 0° starting phase separated by 100 ms. Each tone had a 5-ms rise and fall time and was presented at 80 dB SPL. Target stimuli were identical to comparison stimuli, except for an increase (Δ*t*) in the IOI between one pair of tones. The pause between sequences was 1700 ms.

This task was patterned after the “rhythm discrimination” task in the Test of Basic Auditory Capabilities (TBAC; Watson, 1987; Kidd et al., 2007). Two conditions were included. In the fixed/fixed (*F*/*F*) condition, each tone in the sequence had a frequency of 1000 Hz, and the increased IOI (Δ*t*) always occurred between the 4th and 5th tones. In the variable/variable (V/V) condition, the frequencies of the tones within the sequence randomly varied between 500 and 2000 Hz (in logarithmic spacing), and the position of the increased IOI was variable among the seven possible IOI positions. For this condition, a new random selection of tone frequencies was chosen on each stimulus presentation. For the adaptive tracking procedure, the initial Δ*t* was 30 ms for the *F*/*F* condition and 200 ms for the V/V condition. The step size began at 20% of the initial Δ*t*, and decreased to 10% of the initial Δ*t* after 3 reversals. The IOI could not exceed 400 ms. The order of testing included random selection of fixed/fixed and variable/variable, with the caveat that both conditions were tested before replicates were obtained.

#### Harmonic mistuning

The task was to detect a mistuned component from a harmonic stimulus (e.g., Moore et al., 1985). Comparison stimuli were the sum of 12 harmonically spaced tones generated at a fundamental frequency of 100 or 200 Hz with random phase. The target stimulus was generated by altering the frequency of the 3rd harmonic by Δ*f*, expressed in Hz, with all tones having a newly selected random phase.

Each harmonic component was presented at 80 dB SPL. Stimuli were 400 ms in duration with 40-ms rise-fall times and a 700-ms inter-stimulus interval. In the adaptive tracking procedure, the initial Δ*f* s were 20% of the fundamental frequency. The initial step size of the tracking procedure was a factor of 2, and after three reversals the step size was reduced to a factor of 1.5. The order of testing involved randomly selected fundamental frequency (either 100 or 200 Hz) with the caveat that thresholds were obtained for each fundamental frequency prior to obtaining repeat estimates.

#### Stream segregation

Two tones (or harmonic complexes) were alternated and separated by quiet intervals to form a sequence of triplets: ABA_ABA_ABA_ABA … (Bregman and Campbell, 1971; van Noorden, 1977). Timing was constant, with A, B, and “_” (a silent interval) fixed at 100 ms in duration. The A event was a 250-Hz tone, a 1000-Hz tone, or a 150-Hz harmonic complex consisting of the first 12 harmonics of 150 Hz. All tones (or complexes) had a15 ms rise and fall time. When A was a pure tone, B was also a pure tone, and when A was a harmonic complex, so was B. B began at a frequency (or fundamental frequency) of 1.5 octaves above A. Each subsequent decreasing frequency (or fundamental frequency) of B (*f*_*B*_) was chosen according to the following function:

*f*_*Bn*_ = *f*^(1/1.06)^_*B*(*n−1*)_, where *n* is the triplet number. Each tone was presented at 80 dB SPL.

Listeners were provided two different sets of instructions. In the first block, listeners were told to press a button when they could no longer hear two separate streams (e.g., the fission boundary). In the second block, listeners were told to press a button as soon as they heard a galloping sound (e.g., the “galloping” boundary). This set of instructions was provided to listeners as pilot testing suggested that, at times, listeners had difficulty understanding the first set of instructions. The frequency of the B stimulus when the subjects pressed the button was recorded, and this process was repeated eight times for each set of instructions and frequency. The fission boundary was always tested first, with all eight replicates tested for each randomly selected frequency before a new frequency was tested. The “galloping” boundary was tested second, following a similar randomization procedure.

### Additional auditory tests

Presentation of the stimuli for additional auditory tests, described below, was diotic, rather than monaural. In addition, a high-quality sound card (Digital Audio Labs Card Deluxe) was used instead of the TDT RP2 real-time processor, with a sampling rate of 44,100 Hz.

#### The test of basic auditory capabilities (TBAC)

The TBAC is a battery of auditory tests that has been under development since the early 1980s (see Watson, 1987; Kidd et al., 2007). The version used here was the TBAC-4, obtained from Communication Disorders Technology (CDT), Inc. The test battery includes six tests of auditory discrimination using single tones or groups of tones, and two tests using speech sounds. The eight tests are briefly described below. For additional details see Kidd et al. (2007) and the TBAC information available on the CDT web site (http://comdistec.com/new/TBAC.html).

Trials in each of the subtests, except for subtest 8, are structured in a modified 2AFC format in which a standard stimulus is followed by two test stimuli, one of which is different from the standard. The listeners use a computer keyboard to indicate which test stimulus was different from the standard. Trials are arranged in groups of six, and the level of difficulty is systematically increased from trial to trial, within each group, in logarithmic steps. Eight levels of difficulty are tested over 72 trials, presenting the six easiest levels in the first 36 trials, followed by an increase in difficulty of two log steps for trials 37–72. (This is slightly modified for subtest 7, which uses only five levels and 48 trials.)

The following eight subtests were included. (1) *Single-tone frequency discrimination*: the standard was a 1000-Hz 250-ms tone and frequency increments were used. (2) *Single-tone intensity discrimination:* the standard was a 1000-Hz 250-ms tone and intensity increments were used. (3) *Single-tone duration discrimination*: the standard was a 1000-Hz 100-ms tone and duration increments were used. (4) *Pulse-train discrimination* (rhythm): the standard consisted of six 20-ms pulses (1000-Hz tone) arranged in three pairs, with a 40-ms pause within a pair and a 120-ms pause between pairs. The “different” sequence included an increase in the duration within a pair with a corresponding decrease in the duration between pairs, thus altering the rhythm of the sequence while keeping the total duration constant. (5) *Embedded tone detection:* the standard consisted of a sequence of eight tones of differing frequency with a temporal gap (ranging from 10 to 200 ms) in the middle of the sequence. The “different” sequence had a tone (also ranging from 10 to 200 ms in duration) filling the temporal gap in the middle position. A different sequence of frequencies (ranging from 300 to 3000 Hz) was presented on each trial. The duration of the middle gap or tone was varied to manipulate task difficulty. (6) *Temporal-order discrimination for tones:* the standard was a four-tone pattern consisting of two equal-duration tones (550 and 710 Hz) preceded and followed by a 100-ms 625-Hz tone. The middle tones were presented in reverse order in the “different” interval. The duration of the tones varied from 20 to 200 ms in equi-log steps. (7) *Temporal-order discrimination for syllables*: this subtest is similar to subtest 6, but with consonant-vowel (CV) syllables comprising the sequence instead of tones. The task is to discriminate /fa/-/ta/-/ka/-/pa/ from /fa/-/ka/-/ta/-/pa/. The duration of the syllables was varied (by reducing the vowel duration) from 250 to 75 ms in five steps. (8) *Syllable recognition:* this was a test of the recognition of non-sense CVC syllables in broadband noise. A 3AFC paradigm was used, with foils created by altering the vowel or one of the consonants. Five speech-to-noise ratios (SNRs) were used with decreasing SNRs within each set of five trials. A set of 100 stimuli were presented twice in separate blocks, with a different random order for each block.

#### Test of environmental sound identification

This was a short version of the environmental sound identification (ESI) test described in Kidd et al. (2007). The number of different sounds was reduced from 25 to 20 to keep testing time under an hour. Subjects were asked to identify common non-speech sounds produced by animate or inanimate sources (e.g., a dog barking or a door closing) presented in broadband Gaussian noise. Subjects were initially familiarized with the full set of sounds by listening to them at a favorable SNR with the sound name displayed on the computer screen. During testing, a 3AFC response paradigm was used with the most confusable sounds from the set used as foils. The test consisted of two blocks of 120 trials with each sound presented at each of six overlapping SNRs in each block (8 SNRs in total). Trials were presented in groups of six, with increasing SNRs within each group. The sounds were presented in a different random order in each block of trials, using the highest 6 SNRs in the first block and the lowest 6 SNRs in the second block.

### Visual cognitive-linguistic measures

#### Working-memory tests

Three subtests from a Matlab-based working memory test battery developed by Lewandowsky et al. (2010) were administered. These subtests are described below. Additional details can be found in Lewandowsky et al. (2010). For all tests, there were no time constraints on the recall task at the end of each trial and no feedback was provided. Each test took ~10 min to complete.

***Memory updating.*** At the start of each trial, subjects were presented with a sequence of from 3 to 5 digits. Each digit was surrounded by a square to mark its position on the screen. After all of the digits were presented, the squares remained on the screen and a different sequence of arithmetic operations (addition or subtraction, ranging from +7 to 7) appeared in each of the squares, one at a time. The subject's task was to remember the digits that appeared in each square and then perform the sequence of arithmetic operations presented in each of the squares. The subject was asked to indicate (using the keyboard) the final resulting value in each square after a sequence of from two to six sequential arithmetic operations. The test consisted of 15 trials with a randomly-generated sequence of set size (3–5 co-occurring series of operations) and number of operations (2–6) on each trial.

Because this test was challenging for older adults, some adjustments were made to the procedures to ensure that the task was well-understood, and to make it a bit less challenging. The number of practice trials was increased from two (the default) to four and the time between items (to be added or subtracted) was increased from 250 to 500 ms. The first two practice trials used a 3-s inter-item time to allow the experimenter to explain the required operations during the trial. Also, the default instructions were supplemented with a verbal explanation of the task that included a subject-paced simulated trial using cue cards to present the stimuli.

***Sentence span.*** The “easy” version of the sentence-span task was used for this study. In this task, subjects were presented with an alternating sequence of simple sentences (3–6 words in length) and single letters on the computer screen. Subjects judged whether the sentence was true or false on each presentation, with 4 s allowed for responding. The letters required no response. After from four to eight sentence/letter presentations, subjects were asked to recall the letters in the order they were presented. The test consisted of 15 trials (after three practice trials) with three instances of each number of sentence/letter presentations.

***Spatial short-term memory.*** This test assessed a subject's ability to recall the location of dots (filled circles) in a 10 × 10 grid. On each trial, an empty grid was presented and then a sequence of dots appeared in the grid. Each dot remained on the screen for ~1 s before it was removed and the next dot appeared. From two to six dots were presented on each trial. After all of the dots had been presented (and removed), the subject was asked to indicate the relative position of the dots by touching (or pointing and clicking with a computer mouse) the cells within the grid. This test consisted of 30 trials (6 at each set size).

#### A quick test (AQT)

The AQT was used to provide a measure of cognitive abilities that often decline with age (or due to various types of dementia) (Wiig et al., 2002). The test is designed to measure verbal processing speed, automaticity of naming, working memory, and the ability to shift attention between dimensions of multidimensional visual stimuli. The test consisted of three timed subtests in which subjects named the color and/or the shape of symbols arranged on a page in eight rows of five. Test 1 required subjects to name the color (black, red, blue, or yellow) of colored squares. The second test required subjects to name each shape on a page of black circles, squares, triangles, and lines. The third test included colored shapes (the same shapes and colors used in tests 1 and 2) and subjects were asked to name both the color and the shape. Subjects were told to proceed as fast and as accurately as they could and the total time to complete each subtest was recorded.

#### Text recognition threshold (TRT)

The TRT is a test of the ability to recognize written sentences that are partially obscured by a vertical grating. The Dutch version of the test, developed by Zekveld et al. (2007), was obtained and modified to present English sentences from the revised Speech in Noise (R-SPIN) test (Bilger et al., 1984). No other properties of the test were changed. On each trial, a row of equally-spaced vertical black bars appeared then a sequence of words that form a meaningful sentence, appeared behind (obscured by) the bars. The words appeared sequentially (250 ms per word) and the complete sentence remained on the screen for 3.5 s. The subject's task was to read aloud as much of the sentence as he or she could identify. The difficulty of the task was varied adaptively (based on a subject's performance) by increasing or decreasing the width of the bars (i.e., the percentage of unobscured text). The test consisted of four adaptive runs of 13 trials, with four different sets of R-SPIN predictability-high (PH) sentences. The threshold for each run was computed as the mean percentage of unobscured text on trials 5–13 and the final TRT value was the mean of the four threshold estimates.

### Speech-understanding measures

#### Stimuli

The test battery included a collection of tests to assess the ability to recognize or identify speech under a variety of difficult listening conditions. Four open-set speech-recognition tests utilized the R-SPIN sentences (Kalikow et al., 1977; Bilger et al., 1984). Subsets of these sentences were presented: (1) with 50% time compression; (2) with intermittent interruption; (3) mixed with the original SPIN-test babble; and (4) in quiet. Another set of speech-understanding measures was based on the closed-set identification Coordinate Response Measure (CRM) corpus (Bolia et al., 2000). These measures assessed the ability to identify two key words (color-number coordinates) in a spoken sentence in the presence of a similar simultaneous competing sentence. The competing message was: (1) the same voice as the target message; (2) a voice transposed 6-semitones higher in fundamental-frequency (using STRAIGHT; Kawahara et al., 1999); (3) transposed and time reversed; or (4) not presented (quiet condition).

#### General procedures for speech-understanding measures

As with the other auditory measures, all testing was done in a sound-treated booth that met or exceeded ANSI guidelines for permissible ambient noise for earphone testing (American National Standards Institute, 1999). Stimuli were presented monaurally, using an Etymotic Research ER-3A insert earphone. A disconnected earphone was inserted in the non-test ear to block extraneous sounds. Stimuli were presented by computer using Tucker Davis Technologies System-3 hardware (RP2 16-bit D/A converter, 48,828 Hz sampling rate, HB6 headphone buffer). Each listener was seated in front of a touchscreen monitor, with a keyboard and mouse available.

***Presentation levels.*** For the non-speech auditory measures described previously, audibility of the stimuli could be ensured by judicious selection of stimulus frequencies and levels. For broad-band speech stimuli, however, this is not as easily accomplished. Speech presentation levels were adjusted to ensure that speech information was audible for the older listeners and to provide comparable presentation levels for all listeners. For the older listeners, the long-term spectrum of the full set of stimuli was measured and a filter was applied to shape the spectrum according to each listener's audiogram. The shaping was applied with a 68 dB SPL overall speech level as the starting point, and gain was applied as necessary to each 1/3 octave band to produce speech presentation levels at least 13 dB above threshold from 125 to 4000 Hz. Because this often resulted in relatively high presentation levels, a presentation level of 85 dB SPL (without any spectral shaping) was used for the YNH listeners to minimize level-based differences in performance between groups. Previous work has shown that presentation levels above 80 dB SPL generally lead to somewhat poorer intelligibility, for both uninterrupted speech (e.g., Dubno et al., 2005a,b; Studebaker et al., 1999) and interrupted speech (Wang and Humes, 2010).

#### Measures using the R-SPIN materials

The R-SPIN stimuli are simple sentences, spoken by a male, that consist of five to eight words, ending with a common monosyllabic noun. The R-SPIN materials include 200 PH sentences in which the final word is highly predictable from the prior context, and 200 predictability-low (PL) sentences in which the same final words are presented in a neutral context. Subsets of these sentences were presented in four different stimulus conditions, as described below. The basic procedures and task were the same for all stimulus conditions. On each trial, the word “LISTEN” was presented visually on the monitor, followed by the presentation of a sentence 500 ms later. The subject's task was to type the final word of the sentence using the computer keyboard. Subjects were instructed to make their best guess if they were unsure. The next trial was initiated by either clicking on (with the mouse) or touching a box on the monitor labeled “NEXT.” In addition to responses that were spelled correctly, homophones and equivalent phonetic spellings were scored as correct responses. The four stimulus conditions are described below.

***Time compressed speech.*** A random selection of 100 R-SPIN PL sentences were time-compressed using a 50% time-compression ratio. The time compression was performed using a uniform-compression algorithm, described by Gordon-Salant and Fitzgibbons (1993), applied to the entire sentence. (The time-compressed stimuli were provided by Dr. Gordon-Salant.)

***Interrupted speech.*** Interrupted versions of the R-SPIN sentences were created by digitally replacing portions of each sentence with silence to create a regular pattern of speech fragments, or “glimpses,” throughout the sentence. The glimpse patterns were based on eight equal-duration glimpses of the target word (always the final word in a sentence), with a total glimpsed duration equal to 50% of the total word duration (which ranged from 300 to 600 ms). The onset and offset of each glimpse was smoothed with a 4-ms raised-cosine function to minimize spectral artifacts. The first and last glimpses were always aligned with the beginning and ending of the target word, with the other glimpses equally spaced with a constant pause duration. Consider a total word duration of 320 ms. This word would be interrupted such that 820-ms glimpses (50% of the total word) would be presented with 22.85 ms separating each glimpse. The interruption patterns created to fit the target words were applied to the entire sentence so that a consistent interruption pattern was maintained throughout the sentence, with the location and duration of glimpses determined by the glimpse alignment with the target word. Because of the variation in word duration, the glimpse parameters (8 glimpses and 50% of the word duration) yielded a range of glimpse durations, pause durations, and interruption rates. Because of variation in sentence duration, sentences often began with a single glimpse or silence (pause) duration that was shorter than the value used in the rest of the sentence. Speech-shaped noise was added to each sentence using broadband noise shaped to match the long-term spectrum of the full set of target words. A different randomly chosen section of a 10-s sample of noise was used for each sentence. The duration of the noise sample was adjusted for each sentence, with 250 ms of leading and trailing noise. The speech and noise were mixed at +10 dB SNR measured at the target word (based on rms values). The conditions chosen here were based on prior data for these materials from young and older adults (Kidd and Humes, 2012).

Testing consisted of two blocks of 100 trials. One hundred PL sentences were presented first, followed by 100 PH sentences containing the same set of final words (with a different context sentence).

***Speech in 12-talker babble.*** A different set of PL and PH sentences (100 of each) were then presented with the original R-SPIN 12-talker babble, using a +8 dB SNR. This SNR is common in everyday listening conditions (Pearsons et al., 1977). As with the interrupted speech, the PL sentences were presented prior to the PH sentences in two 100-trial blocks.

***Speech in quiet.*** The final test with the R-SPIN materials presented 100 intact PL sentences in quiet. The sentences were the 100 that had not been presented in the time-compression test. To provide a break from the R-SPIN materials, subjects completed the AQT and the Speech, Spatial, and Qualities of Hearing Scale (SSQ) measures, described below, after the babble condition before returning to the R-SPIN materials for this test.

#### Measures using the CRM

The CRM corpus (Bolia et al., 2000) consists of a collection of sentences spoken by four male and four female talkers. All sentences are of the form “Ready [call sign] go to [color] [number] now.” There are eight call signs (arrow, baron, charlie, eagle, hopper, laker, ringo, tiger), four colors (blue, green, red, white) and eight numbers (1–8) spoken in all 256 combinations by each talker. The test battery utilized a single male voice presented either in the original form or transformed (time reversed and/or transposed in fundamental frequency by 6 semitones). These conditions represent a subset of those described in Lee and Humes (2012). On each trial, two different sentences were presented simultaneously and the task was to listen to the voice that said “baron” as the call sign (always the original unaltered voice) and report the color and number spoken by that voice. Each trial began with the word “LISTEN” presented visually on the display, followed 500 ms later by presentation of the sentences. After each presentation, subjects responded by touching (or clicking with a mouse) virtual buttons on a touch screen display to indicate whether they heard the “baron” call sign (which was always spoken), and, if so, to indicate the color and number spoken by the same talker. All four colors and all eight numbers were included on the response display which remained in view throughout each block of trials. The next trial was initiated by either clicking on (with the mouse) or touching a box on the monitor labeled “OK.” All trial blocks consisted of 32 trials. The sequence of trial blocks was as follows: (1) two trial blocks with no competing sentence, for familiarization with the task; (2) one practice trial block with examples of each of the different listening conditions; (3) four trial blocks of simultaneous competing sentences in the same male voice (unaltered); (4) four trial blocks with a 6-semitone shift of the fundamental frequency of the target or competing voice (50% each within each block); (5) a second set of four trial blocks with simultaneous competing sentences in the same voice; and (6) four trial blocks in which the competing sentences were both time-reversed and transposed (F0 by 6 semitones).

#### Vowel-sequence identification

This task used four speech stimuli that consisted of the center 40-ms of vowels produced naturally in a /p/-vowel-/t/ context by a male talker (Fogerty et al., 2010). The first vowel presented was randomly selected from the four alternatives and presented randomly to either the right or left ear. After a variable stimulus onset asynchrony (SOA), the second vowel, randomly selected from the remaining three vowels, was presented to the opposite ear. The SOA was randomly selected from a set of six SOAs (120–170 ms in 10-ms steps) to encompass the linear portion of the psychometric function relating identification performance to SOA, The subject's task was to identify the vowel pair presented, and only those responses identifying the two vowels in the correct sequence from the choices presented on the PC touchscreen were scored as correct. Responses were collapsed across SOA values, and overall percent-correct performance was recorded. A total of 144 trials were presented with equal distribution of left-ear leading and right-ear leading trials, SOAs, and the 12 possible vowel pairs.

### Speech, spatial, and qualities of hearing scale (SSQ)

The SSQ is a questionnaire developed by Gatehouse and Noble (2004) to measure auditory disability through self-report of various aspects of hearing in a variety of common settings. The questionnaire covers speech-understanding difficulties with different competing backgrounds, as well as aspects of spatial hearing and sound quality. Version 3.1.2b was used for this study. This version included 14 questions about speech understanding, 17 questions about spatial hearing and sound localization, and 22 questions about sound quality (including sound segregation, music and voice identification, and sound source identification). Because the vast majority of subjects were not hearing-aid wearers, the approach described by Singh and Pichora-Fuller (2010) was followed in which 7 of the 53 SSQ items related to hearing aids were eliminated prior to scoring (Qualities subscale items 15, 16, 17, 20, 21, and 22; Spatial subscale item 14). All scales are arranged such that higher scores indicate fewer difficulties.

## Results and discussion

### Reliability of measures

As noted, the reliability of the various test measures included in this study was evaluated in a subsample of 31 of the 98 older adults who were tested twice with the entire test battery. Table 1 displays the means and standard deviations for the test and retest conditions. The mean test-retest interval from the beginning of Session 1-test to the beginning of Session 1-retest was 98.8 days with a range of 78–129 days. There were a total of 9 sessions required for the full test battery and the order of the sessions, as well as the tests within each session, were identical in both the test and retest conditions.

**Table 1:** **Means (M) and standard deviations (SD) for the test and retest conditions for a group of 31 older adults**.

**Measure**	***N***	**Test *M* (*SD*)**	**Retest *M* (*SD*)**	***p***	***r***
Info mask 500 Hz MB same	31	90.8 (9.2)	87.1 (10.8)	0.003	0.82^*^
Info mask 500 Hz MB diff	31	79.8 (13.9)	72.1 (15.4)	<0.001^*^	0.79^*^
Info mask 1 kHz MB same	31	90.3 (8.0)	85.5 (9.4)	<0.001^*^	0.77^*^
Info mask 1 kHz MB diff	31	75.3 (17.6)	66.5 (17.3)	<0.001^*^	0.86^*^
Modulation detection 5 Hz	30	−16.6 (3.5)	−17.9 (2.9)	0.01	0.59^*^
Modulation detection 20 Hz	30	−20.5 (2.6)	−21.2 (2.4)	0.08	0.64^*^
Modulation detection 60 Hz	30	−18.7 (2.3)	−19.1 (2.1)	0.27	0.38
MDI 5 Hz unmod interferer	31	−17.8 (4.0)	−19.0 (4.1)	0.02	0.80^*^
MDI 5 Hz mod interferer	31	−6.2 (2.1)	−6.4 (2.2)	0.62	0.51
MDI 10 Hz unmod interferer	31	−22.5 (3.2)	−22.6 (3.1)	0.95	0.66^*^
MDI 10 Hz mod interferer	31	−7.8 (3.6)	−7.6 (2.6)	1.78	0.70^*^
MDI 20 Hz unmod interferer	31	−22.8 (3.1)	−23.1 (3.0)	0.36	0.75^*^
MDI 20 Hz mod interferer	31	−9.7 (3.2)	−10.2 (2.8)	0.45	0.43
MLD 250 Hz S_o_	29	70.2 (2.7)	69.4 (3.1)	0.21	0.43
MLD 250 Hz S_π_	29	61.8 (4.7)	61.7 (6.2)	0.91	0.63^*^
MLD 500 Hz S_o_	29	69.5 (2.4)	68.9 (2.2)	0.18	0.56
MLD 500 Hz S_π_	29	59.6 (3.2)	58.5 (3.2)	0.03	0.67^*^
Anisochron var-var (ms)	31	87.3 (47.7)	74.6 (35.6)	0.12	0.47
Anisochron fixed-fixed (ms)	31	16.8 (6.4)	14.3 (4.5)	0.004	0.74^*^
Harm mistuning 100 Hz (Hz)	30	13.1 (9.4)	9.2 (8.0)	<0.001^*^	0.88^*^
Harm mistuning 200 Hz (Hz)	30	13.6 (10.0)	8.9 (4.2)	0.003	0.65^*^
Stream seg 1 150 Hz (Hz)	31	193.8 (19.9)	191.9 (18.1)	0.55	0.62^*^
Stream seg 1 250 Hz (Hz)	31	327.1 (46.2)	316.7 (38.7)	0.20	0.49
Stream seg 1 1 kHz (Hz)	31	1211.9 (170)	1183.3 (144)	0.38	0.37
Stream seg 2 150 Hz (Hz)	31	232.0 (68.8)	207.4 (47.2)	0.02	0.63^*^
Stream seg 2 250 Hz (Hz)	31	375.6 (117.7)	341.6 (75.9)	0.03	0.69^*^
Stream seg 2 1 kHz (Hz)	31	1437.5 (416)	1299.6 (308)	0.04	0.55
TBAC 6avg (%)	29	75.4 (7.7)	79.1 (5.6)	<0.001^*^	0.76^*^
Environmental sounds (%)	31	60.5 (5.4)	64.9 (4.9)	<0.001^*^	0.50
Memory updating (%)	29	46.9 (19.2)	52.1 (19.8)	0.05	0.83^*^
Sentence span (%)	29	50.9 (15.0)	53.3 (15.1)	0.07	0.83^*^
Spatial STM (%)	29	72.0 (7.0)	73.3 (6.4)	0.37	0.69^*^
AQT color (s)	31	24.0 (6.5)	23.7 (6.3)	0.70	0.92^*^
AQT shape (s)	31	29.4 (8.0)	28.3 (6.7)	0.21	0.87^*^
AQT color + shape (s)	31	54.7 (14.8)	55.3 (16.5)	0.56	0.89^*^
TRT (% unmasked)	29	38.6 (4.5)	39.3 (4.6)	0.07	0.91^*^
SPIN-PL time comp (%)	31	72.7 (16.1)	77.5 (14.2)	0.007	0.81^*^
SPIN-PL interrupted (%)	31	43.6 (16.1)	52.0 (16.7)	0.001	0.68^*^
SPIN-PH interrupted (%)	31	98.0 (18.5)	84.0 (15.4)	0.01	0.81^*^
SPIN-PL babble (%)	31	60.0 (15.1)	66.9 (16.6)	0.003	0.68^*^
SPIN-PH babble (%)	31	94.1 (8.3)	91.8 (14.2)	0.36	0.46
CRM no competition (%)	31	99.3 (1.2)	99.4 (1.3)	0.93	0.17
CRM same talker comp (%)	31	23.6 (7.6)	22.7 (5.8)	0.58	0.46
CRM 6 ST Fo shift (%)	31	34.9 (11.4)	37.6 (14.1)	0.02	0.88^*^
CRM 6 ST Fo shift + rev (%)	31	83.8 (22.5)	87.6 (19.0)	0.09	0.83^*^
Vowel sequence identif (%)	31	52.7 (25.3)	58.2 (25.3)	0.008	0.91^*^
SSQ speech scale	30	7.1 (1.5)	7.0 (1.5)	0.41	0.70^*^
SSQ spatial scale	30	7.7 (1.5)	7.6 (1.6)	0.70	0.83^*^
SSQ qualities scale	30	8.2 (1.2)	8.1 (1.2)	0.77	0.83^*^
SSQ overall score	30	7.7 (1.2)	7.6 (1.3)	0.55	0.80^*^

*The actual sample size varied slightly across measures and is indicated in the second column (N). The p-value for paired-sample t-tests of these two means is also provided as is the Pearson-r correlation coefficient between test and retest. In both cases, significant t-test p-values and r-values are marked with an asterisk. Given 50 measures, the criterion p-value for statistical significance using Bonferroni adjustment for multiple comparisons is p < 0.001 (i.e., 0.05/50). Units for each measure are dB unless indicated otherwise*.

Table 1 also provides the significance levels (*p*) for paired-sample *t*-tests, for comparisons of performance from test to retest, as well as the Pearson-*r* correlation coefficients between test and retest. Given 50 variables in Table 1, a conservative Bonferroni adjustment for multiple comparisons was applied when interpreting the significance of both the *t*-tests and the correlations. As a result, the criterion for statistical significance is a *p*-value for both the *t*-statistic and the correlation coefficient that is less than 0.001 (i.e., 0.05/50). Using this criterion for statistical significance, significant *t*-statistics and Pearson correlations are marked with an asterisk in Table 1. For six of the measures listed in Table 1, there is a significant difference in performance from test to retest with five of the six measures (3 informational-masking measures, one harmonic-mistuning measure, and environmental sound identification) improving from test to retest and one measure (TBAC) showing worse performance at retest. These differences in mean performance were not as important as the consistency of the measures from test to retest among the group of 31 older adults. This consistency is, for the most part, represented by the test-retest correlations. A test-retest correlation of *r* = 0.60 was adopted as the minimum acceptable test-retest correlation and this corresponds well to the boundary between the statistically significant and non-significant correlation coefficients. Application of this criterion for minimally acceptable test-retest correlation resulted in the initial elimination of the 13 measures in Table 1 for which the correlation was not significant. Only one significant correlation (modulation detection at 5 Hz, *r* = 0.59) was eliminated using a criterion of *r* ≥ 0.6. For the 14 measures with test-retest correlations less than 0.60, scatterplots of test and retest scores were reviewed. In two of these 14 cases, correlations were low because of ceiling effects in the data (SPIN-PH in babble and CRM in quiet) and these variables were retained for subsequent analyses. It should be noted that the SPIN-PL scores in quiet were very high during the test condition and would have likely shown a low test-retest correlation as well, but this measure was inadvertently omitted from the retest condition. Another of the 14 measures eliminated initially, CRM scores for the same-talker competition, was restored for subsequent analyses following examination of the test-retest scatterplots. This low test-retest correlation (*r* = 0.46) was due to two outliers among the 31 subjects who experienced an unusually large improvement in scores (15–20% points) from test to retest. Without these two data points the test-retest correlation for this measure exceeded 0.80. Finally, the low test-retest correlation for ESI test was examined and it too was found to exceed the *r* = 0.60 cutoff with two outliers removed (*r* = 0.50 before removal and 0.65 after removal). Although the impact of the outliers was not as great as with CRM, the ESI test was retained because of the previous demonstration of its reliability (Kidd et al., 2007) and because it is the only measure of this particular auditory ability in the test battery. In the end, a total of 10 measures were eliminated from further analyses due to poor test-retest reliability and all of these were auditory non-speech measures.

### Group data: older adults vs. young normal-hearing adults

Table 2 provides the means and standard deviations for 41 measures, the 40 reliable measures identified in the previous section plus the SPIN-PL score in quiet, for the group of 98 older adults and 27 younger adults. Given 41 measures, a conservative Bonferroni adjustment of the criterion for statistical significance yields a criterion *p*-value of 0.00122 (i.e., 0.05/41). Using this criterion, the *p*-values for significant independent-groups *t*-tests have been marked in Table 2 with asterisks. Of the 41 measures in Table 2, 15 revealed significant differences between young and older adults; 12 showing older adults performing worse than young adults and 3 showing older adults performing better than young adults. (The latter tests are marked with a plus sign by the older group's score.) For the three measures for which older adults outperformed young adults, two (TRT, SPIN-PH interrupted) made use of high-context sentences. Superior performance of older adults on such materials is consistent with superior verbally-based cognitive scores in older adults (e.g., Salthouse, 2010) and is a phenomenon that has been observed frequently, but not universally, with the SPIN-PH test materials [see review in Humes et al. (2007), and note the slightly worse performance of older listeners with interrupted PH and PL materials observed by Kidd and Humes (2012)]. Superior performance of older adults on the SPIN-PH materials was not observed here for the babble test conditions, but this could be due, in part, to the ceiling effects observed in this condition. The lone remaining case in Table 2 of superior performance of older adults was for modulation detection for a 20-Hz modulated broad-band noise and it is unclear why the older adults outperformed the young adults for this task.

**Table 2:** **Means (M) and standard deviations (SD) for the groups of young (*N* = 27) and older (*N* = 98) adults**.

**Measure**	**Young *M* (*SD*)**	**Older *M* (*SD*)**	***t* (*df* = 123)**	***p***
Info mask 500 Hz MB same	86.8 (8.8)	90.3 (9.4)	−1.77	0.08
Info mask 500 Hz MB diff	63.9 (11.8)	79.8 (14.2)	−5.33	<0.001^*^
Info mask 1 kHz MB same	84.4 (9.8)	88.2 (9.5)	−1.83	0.07
Info mask 1 kHz MB diff	58.9 (13.6)	74.7 (16.2)	−4.63	<0.001^*^
Modulation detection 20 Hz	−16.8 (2.7)	−20.6 (2.7)^+^	6.37	<0.001^*^
MDI 5 Hz unmod interferer	−16.4 (5.3)	−18.3 (4.1)	1.96	0.05
MDI 10 Hz unmod interferer	−21.2 (3.8)	−22.8 (3.1)	2.27	0.02
MDI 10 Hz mod interferer	−8.1 (3.0)	−7.8 (3.5)	−0.40	0.69
MDI 20 Hz unmod interferer	−21.8 (3.0)	−23.2 (3.6)	1.84	0.07
MLD 250 Hz S_π_	57.4 (4.9)	62.1 (7.5)	−3.05	0.003
MLD 500 Hz S_π_	56.6 (4.4)	60.5 (6.1)	−3.12	0.002
Anisochron fixed-fixed (ms)	16.3 (9.1)	16.2 (8.3)	0.05	0.96
Harm mistuning 100 Hz (Hz)	10.6 (7.8)	11.5 (8.2)	−0.54	0.59
Harm mistuning 200 Hz (Hz)	10.8 (7.3)	14.0 (10.9)	−1.44	0.15
Stream seg 1 150 Hz (Hz)	205.7 (21.2)	192.6 (18.3)	3.19	0.002
Stream seg 2 150 Hz (Hz)	224.9 (35.2)	224.0 (61.7)	0.08	0.94
Stream seg 2 250 Hz (Hz)	371.4 (61.7)	366.9 (100.8)	0.22	0.83
TBAC 6avg (%)	82.9 (6.0)	76.1 (8.3)	3.93	<0.001^*^
Environmental sounds (%)	65.59 (6.7)	60.88 (6.4)	3.37	<0.001^*^
Memory updating (%)	75.2 (10.4)	51.6 (20.5)	5.78	<0.001^*^
Sentence span (%)	70.7 (12.6)	53.5 (16.5)	5.05	<0.001^*^
Spatial STM (%)	84.9 (4.8)	72.7 (7.1)	8.44	<0.001^*^
AQT color (s)	19.4 (2.6)	23.0 (5.1)	−4.99	<0.001^*^
AQT shape (s)	23.1 (3.1)	29.7 (7.1)	−7.03	<0.001^*^
AQT color + shape (s)	48.1 (6.7)	55.4 (14.7)	−3.69	<0.001^*^
TRT (% unmasked)	42.9 (2.8)	38.5 (4.6)^+^	4.83	<0.001^*^
SPIN-PL quiet (%)	95.7 (6.5)	92.4 (11.6)	1.42	0.16
SPIN-PL time comp (%)	85.3 (9.9)	69.6 (19.3)	4.07	<0.001^*^
SPIN-PL interrupted (%)	35.9 (15.8)	43.6 (16.1)	−2.24	0.03
SPIN-PH interrupted (%)	59.2 (22.3)	78.5 (18.8)^+^	−4.52	<0.001^*^
SPIN-PL babble (%)	67.0 (13.9)	60.3 (17.8)	1.81	0.07
SPIN-PH babble (%)	91.9 (11.4)	94.4 (10.0)	−1.12	0.26
CRM no competition (%)	96.5 (16.8)	99.3 (1.3)	−1.67	0.10
CRM same talker comp (%)	26.1 (8.4)	23.4 (6.8)	1.74	0.08
CRM 6 ST Fo shift (%)	43.0 (15.2)	34.3 (11.8)	3.17	0.002
CRM 6 ST Fo shift + rev (%)	92.9 (16.3)	82.9 (25.4)	1.95	0.05
Vowel sequence identif (%)	56.6 (20.5)	53.3 (24.1)	0.63	0.53
SSQ speech scale	8.2 (0.8)	6.9 (1.6)	3.81	<0.001^*^
SSQ spatial scale	7.8 (1.5)	7.7 (1.5)	0.47	0.64
SSQ qualities scale	8.7 (1.1)	8.2 (1.1)	1.90	0.06
SSQ overall score	8.2 (1.0)	7.6 (1.2)	2.37	0.02

*The t-value (t) and p-value (p) for independent-sample t-tests of these two means is also provided. Significant t-values are marked with an asterisk. Given 39 measures, the criterion p-value for statistical significance using Bonferroni adjustment for multiple comparisons is p < 0.00122 (i.e., 0.05/41). Units for each measure are dB unless indicated otherwise. Tests for which the older group performed significantly better than the younger group are indicated by a plus sign by the older groups' score*.

For the 12 measures in Table 2 for which older adults performed significantly worse than young adults, six of these are cognitive measures; the three working-memory tasks and the three AQT verbal speed-of-processing measures. For such processing-based measures of cognitive function, steady declines in performance throughout adulthood have been well-documented [see review by Salthouse (2010)] and the present data are entirely consistent with the literature. Of the remaining six measures for which the older adults performed significantly worse than the young adults, three involved non-speech auditory psychophysical measures (two informational-masking measures and the TBAC), one was a non-speech sound identification test (ESI), one involved speech-recognition performance (SPIN-PL, time compressed), and one was the speech subscale of the SSQ. The results for the SSQ are generally consistent with the effects of age and hearing loss observed for this self-report measure previously (Gatehouse and Noble, 2004; Singh and Pichora-Fuller, 2010; Banh et al., 2012). Likewise, the group differences for the time-compressed SPIN-PL items are similar to the results observed by Gordon-Salant and Fitzgibbons (1993) for the same test materials. In general, the performance by older listeners on psychoacoustic tasks observed here is consistent with earlier work. Prior research with the TBAC (Christopherson and Humes, 1992) has shown poorer performance on some tasks (primarily temporal-processing measures and speech tests), and many other investigations with psychoacoustic tasks have shown that older listeners often perform as well as younger listeners with simpler stimuli and non-temporal tasks, but are likely to have greater difficulty with temporal tasks and more complex stimuli (see Fitzgibbons and Gordon-Salant, 2010, for a review).

It is interesting that for the 11 measures of speech-understanding included in Table 2, only one, the time-compressed SPIN-PL test, revealed significantly worse performance for the older adults, despite the presence of varying degrees of high-frequency hearing loss in the older adults. It is important to recall, however that the speech stimuli used in each of these speech-understanding measures had been spectrally shaped in this study to minimize the contributions of inaudibility. Nonetheless, the cochlear pathology presumed to underlie the hearing loss is still present, but, along with age, seems to have little impact on most of the measures of speech-understanding included in this study, with the exception of time-compressed low-context sentences.

### Individual differences among older adults

As a first step in examining individual differences in performance among the 98 older adults in this study, factor analysis was used to reduce the redundancy among the various sets of variables. There were three basic sets of variables in this study in which most of the measures could be placed: (1) auditory non-speech psychophysical measures; (2) cognitive/linguistic-processing measures; and (3) speech-understanding measures. There were 18 auditory non-speech psychophysical measures that, based on the information presented previously in Table 1, were considered sufficiently reliable for further analyses. To these 18 variables were added two measures of average hearing loss: (1) pure-tone average (PTA), which was the mean hearing loss at 500, 1000, and 2000 Hz in the test ear; and (2) high-frequency pure-tone average (HFPTA), which was the mean hearing loss at 1000, 2000, and 4000 Hz in the test ear. These 20 non-speech auditory measures were subjected to a principal-components factor analysis in an effort to reduce redundancy among the variables and, possibly, depending on the outcome of these analyses, also minimize collinearity among this set of variables. These factor analyses were exploratory and empirically motivated, not confirmatory or theoretically motivated. The primary objective was simply to capture the greatest amount of variance among this set of 20 measures and represent that variance with a smaller number of factors. In the initial factor analysis, and all subsequent factor analyses, the analysis made use of oblique rotation of factors (Promax rotation; κ = 4; Gorsuch, 1983) and the between-factor correlation matrix was examined if more than one factor emerged. If any between-factor correlations exceeded a value of 0.40, then the set of correlated factor scores were saved for each subject for a subsequent second-order factor analysis. If the initial factor analysis failed to generate any between-factor correlations above 0.40, then an uncorrelated or orthogonal fit was considered appropriate and the set of orthogonal factor scores was saved for each subject.

For the auditory non-speech psychophysical measures, the initial factor analysis showed low communalities for three measures, Modulation Detection at 20 Hz, the TBAC, and Stream Segregation-1 for a 150-Hz fundamental. This was reflected, in all cases, in low component weights for all factors included in the solution. As a result, these three variables were dropped and the factor analysis was repeated for the set of 17 auditory non-speech psychophysical measures. A good solution was then obtained, accounting for 74.6% of the variance with five factors. Low between-factor correlations (*r* < 0.40) in the initial oblique rotation supported the subsequent use of orthogonal rotation. Communalities were all in excess of 0.43 with most (13 of 17) ≥ 0.70 and the KMO sampling-adequacy statistic was reasonably high (0.67). The component weights of each of the 17 auditory psychophysical measures on each of the five resulting rotated (varimax) orthogonal factors are shown in Table 3. Based on the patterns of these component weights Factors 1 through 5, respectively, were interpreted and labeled as follows: (1) Informational Masking (InfMask); (2) Signal Envelope Processing (ModDet); (3) Hearing Loss and Harmonic Mistuning (HLoss_HM; (4) Dichotic Signal Detection (DicSigDet); and (5) Stream Segregation (StrmSeg). For the HLoss_HM factor, the component weights for the hearing loss measures were clearly stronger than those for the harmonic-mistuning measures. The correlations between the four independent variables underlying this factor (two PTAs and two measures of harmonic mistuning, each with a different fundamental frequency) ranged from *r* = 0.28–0.35, suggesting about 10% common variance between hearing loss and harmonic mistuning.

**Table 3:** **Component weights for each psychoacoustical measure on each of the five orthogonal principal components identified via factor analysis**.

**Measure**	**Factor 1**	**Factor 2**	**Factor 3**	**Factor 4**	**Factor 5**
Info mask 500 Hz MB same	**0.890**	0.064	0.092	0.048	−0.044
Info mask 500 Hz MB diff	**0.894**	0.097	0.088	−0.031	0.013
Info mask 1 kHz MB same	**0.869**	0.135	−0.013	0.056	0.077
Info mask 1 kHz MB diff	**0.917**	0.051	0.059	0.067	0.044
MDI 5 Hz unmod interferer	0.079	**0.795**	0.058	0.110	0.208
MDI 10 Hz unmod interferer	0.047	**0.904**	−0.061	0.121	0.002
MDI 10 Hz mod interferer	0.023	0.284	**−0.539**	0.147	−0.192
MDI 20 Hz unmod interferer	0.044	**0.868**	−0.118	0.099	−0.107
MLD 250 Hz S_π_	0.038	0.221	0.146	**0.878**	0.036
MLD 500 Hz S_π_	0.098	0.070	0.160	**0.887**	−0.006
Anisochron fixed-fixed (ms)	0.262	**0.564**	0.234	−0.033	−0.074
Harm mistuning 100 Hz (Hz)	0.367	0.253	**0.519**	0.159	−0.077
Harm mistuning 200 Hz (Hz)	0.325	0.306	**0.640**	−0.005	−0.114
Stream seg 2 150 Hz (Hz)	0.003	0.008	0.096	0.034	**0.975**
Stream seg 2 250 Hz (Hz)	0.061	0.014	0.061	0.023	**0.966**
Pure-tone average (PTA)	−0.007	−0.029	**0.744**	**0.465**	0.142
High-frequency PTA	−0.079	−0.055	**0.770**	**0.418**	0.114

*Weights >0.4 are shown in bold typeface*.

Next, the six cognitive measures (three measures of working memory and the three measures of verbal speed of processing from the AQT) were subjected to a similar analysis. A good solution was obtained (KMO sampling adequacy statistic = 0.79; all communalities ≥ 0.62) and 76.5% of the variance was explained by two moderately correlated (*r* = −0.44) factors. Table 4 shows the rotated oblique component (pattern matrix) weights for the initial factor solution. The two factors were interpreted as working memory and verbal speed of processing based on the pattern of component weights across factors. A second-order principal-components factor analysis was then performed on these two correlated factor scores and a single global cognitive processing factor resulted (KMO = 0.50; communalities = 0.72; 72.1% of the variance accounted for). This single global cognitive-processing factor was labeled CogProc_Global and saved for all subjects.

**Table 4:** **Component weights from the pattern matrix for each cognitive measure on each of the two oblique principal components identified via factor analysis**.

**Measure**	**Factor 1**	**Factor 2**
Memory updating (%)	−0.171	**0.798**
Sentence span (%)	−0.341	**0.575**
Spatial STM (%)	0.260	**0.947**
AQT color (s)	**0.914**	0.078
AQT shape (s)	**0.924**	0.015
AQT color + shape (s)	**0.898**	−0.013

Weights >0.4 are shown in bold typeface.

Next, the 10 speech-understanding measures shown in Table 5 were subjected to a principal-components factor analysis. A good solution was obtained with two factors and the resulting component weights for the pattern matrix of the oblique-rotated solution are shown in Table 5 (KMO sampling adequacy statistic = 0.84; all communalities ≥ 0.54). A total of 67.8% of the variance was explained by two moderately correlated (*r* = 0.50) factors. Based on the pattern of weights in Table 5, the two moderately correlated factors were interpreted as open-set recognition, with mainly the R-SPIN tests loading heavily on this factor, and closed-set speech identification, with CRM and vowel-sequence identification tests loading on this factor. A second-order principal-components factor analysis was then performed on these two correlated factor scores and a single global speech-understanding factor resulted (KMO = 0.50; communalities = 0.75; 74.8% of the variance accounted for). This single global speech-understanding factor was labeled SpeechUnd_Global and saved for all subjects.

**Table 5:** **Component weights from the pattern matrix for each speech-understanding measure on each of the two oblique principal components identified via factor analysis**.

**Measure**	**Factor 1**	**Factor 2**
SPIN-PL quiet (%)	**0.903**	−0.153
SPIN-PL time comp (%)	**0.920**	−0.014
SPIN-PL interrupted (%)	**0.687**	0.167
SPIN-PH interrupted (%)	**0.825**	0.136
SPIN-PL babble (%)	**0.903**	−0.040
SPIN-PH babble (%)	**0.922**	−0.137
CRM same talker comp (%)	−0.262	**0.872**
CRM 6 ST Fo shift (%)	0.193	**0.673**
CRM 6 ST Fo shift + rev (%)	**0.530**	0.310
Vowel Sequence Identif (%)	0.112	**0.670**

*Weights >0.4 are shown in bold typeface*.

Finally, the three scales of the SSQ were subjected to a principal-components factor analysis. A single factor emerged, accounting for 72.8% of the variance, with a good KMO sampling-adequacy statistic (0.67) and all communalities exceeding 0.67. This factor score was saved for all subjects at SSQ_Global.

Ultimately, the focus of this study was to explain individual differences among older adults in aided speech understanding (SpeechUnd_Global) or everyday self-reported speech perception (SSQ_Global) using various predictor variables. The large set of non-speech psychoacoustic predictor variables was reduced to five orthogonal factor scores and the six cognitive measures were reduced to one global factor score (CogProc_Global). ESI and TRT scores were added as additional potential predictors that were not necessarily represented well by any of the other predictor variables, but could be of significance. Correlations among the predictors and the two speech measures are shown in Table 6. Although Age is not a direct causal factor contributing to speech understanding, it is included in Table 6 to show its correlation with other variables that may be causal factors in the observed relation between age and speech understanding. All but two of the measures in Table 6 have significant correlations with SpeechUnd_Global, with the highest correlations for Age, Cognition, and ESI (all greater than 0.5). Only three measures have significant correlations with SSQ_Global (HLoss_HarmMis, CogProc_Global, and ESI).

**Table 6:** **Correlation matrix of selected predictor variables and global speech understanding measures**.

	**Age**	**Inf Mask**	**Mod Det**	**HLoss_HM**	**StrmSeg**	**DichSigDet**	**Cog_Global**	**ESI**	**SpeechUnd Global**	**SSQ Global**
TRT	−0.239	−0.132	−0.197	−0.269^*^	0.091	−0.360^*^	0.522^*^	0.073	0.466^*^	0.229
Age		0.123	−0.128	0.510^*^	−0.067	0.216	−0.375^*^	−0.501^*^	−0.515^*^	−0.086
InfMask			0.000	0.000	0.000	0.000	−0.185	−0.098	−0.376^*^	0.054
ModDet				0.000	0.000	0.000	−0.248	−0.147	−0.113	−0.203
HLoss_HM					0.000	0.000	−0.301^*^	−0.406^*^	−0.433^*^	−0.351^*^
StrmSeg						0.000	−0.093	−0.183	0.006	−0.143
DichSigDet							−0.254	−0.257	−0.335^*^	−0.151
Cog_Global								0.398^*^	0.543^*^	0.390^*^
ESI									0.535^*^	0.269^*^
SpeechUnd Global										0.263^*^

* *p < 0.01*.

**Figure 2:**
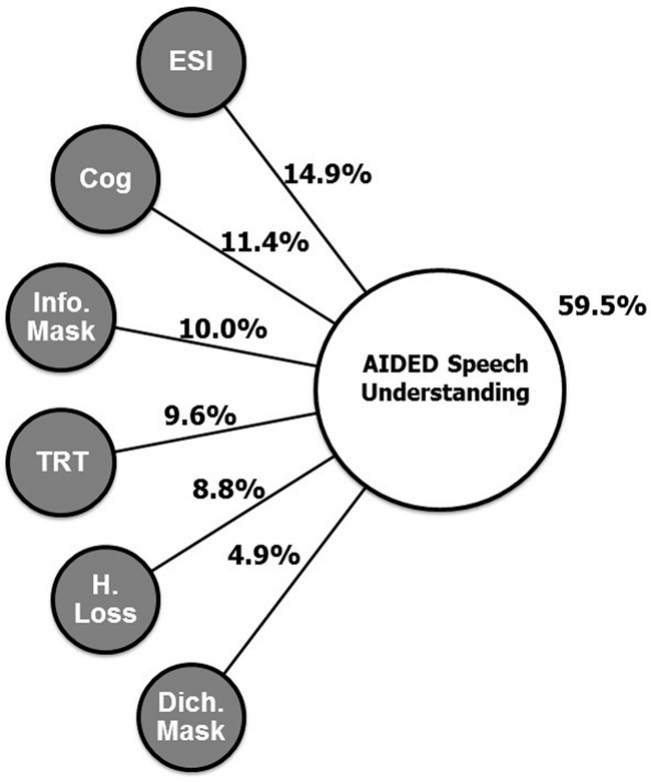
**Schematic illustration of the best-fitting regression model from the dominance analysis showing the total variance explained for aided speech understanding (59.5%) and the relative proportions of variance associated with each predictor variable.** See text for further descriptions of the predictors and the analyses.

To determine the relative contributions of the various measures in accounting for speech understanding (SpeechUnd_Global), a dominance analysis (Azen and Budescu, 2003; Budescu and Azen, 2004) was performed with six of the nine predictor variables in Table 6. Two of the five psychoacoustic variables (ModDet and StrmSeg) were omitted because their correlations with SpeechUnd_Global were non-signifiant, and quite low. Age was not included in the dominance analysis, because it is not a direct causal factor, and because it did not account for any significant variance in SpeechUnd_Global that was not accounted for by the other variables in Table 6. Results of the dominance analysis are shown in Table 7. Dominance analysis provides a measure of importance for each predictor variable, which is the average amount of variance accounted for by each variable when entered into the regression equation alone and after each of the possible subsets of the other predictor variables, with subset size (*k* in Table 7) ranging from 0 to 5 in the present case. A rescaled version of this importance measure is also provided by expressing the average variance for each variable as a percentage of the total variance accounted for by the full set of variables (59.5% in this case). Given the average test-retest reliability of the measures that make up the speech understanding factor (about *r* = 0.8), the maximum variance that one could expect to account for in this case would be 64% (0.8^2^). Thus, these measures account for roughly 93% (59.5/64) of the “systematic variance” in speech understanding. As seen in Table 7, ESI has the highest rescaled dominance value (25%), followed by Cognition (19.2%), with TRT, InfMask, and HLoss_HM close behind (14.8–16.8%), and a much smaller value for the Dichotic measure (8.2%). This model is represented in Figure 2. The general dominance of ESI falls short of conditional dominance due to a single value of *k* (*k* = 0) at which another variable (Cognition) accounts for slightly greater variance. [See Azen and Budescu (2003), for a discussion of complete, conditional, and general dominance.]

**Table 7:** **Dominance analysis for prediction of SpeechUnd_Global**.

***k***	**Cog_Global**	**ESI**	**TRT**	**Inf Mask**	**Hloss_HM**	**DichSig Det**
0	0.295	0.286	0.217	0.141	0.188	0.112
1	0.179	0.198	0.135	0.114	0.123	0.068
2	0.106	0.146	0.088	0.097	0.084	0.044
3	0.060	0.112	0.062	0.088	0.060	0.031
4	0.031	0.086	0.043	0.082	0.043	0.022
5	0.014	0.063	0.029	0.078	0.029	0.014
General dominance	0.114	0.149	0.096	0.100	0.088	0.049
Rescaled dominance	19.20	24.97	16.08	16.81	14.75	8.17

*R^2^-values resulting from the entry of each variable in the presence of each possible subset of the other variables are computed, and the average R^2^-values across subsets of size k = 0 through 5 (the total number of variables −1) for each variable are shown in the table. General dominance is the average R^2^-value across all levels of k. Rescaled dominance is the general dominance value rescaled as a percentage of the total variance accounted for by the full model*.

The importance of ESI in accounting for Speech Understanding is consistent with earlier findings by Kidd et al. (2007), who found that a similar ESI test was the only one of 16 non-speech measures to have its strongest loading on a factor defined by three speech-recognition measures (and the ESI measure). The present finding provides further support for the notion of a general familiar-sound recognition ability that is not specific to speech, and also reinforces the idea that this ability is distinct from a general cognitive or intellectual ability. It should also be noted that the other auditory measures in this test battery found to be related to speech understanding would not be considered to be measures of temporal or spectral resolution; areas of focus in many prior studies. Rather, they are largely measures that involve higher level processes such as selective auditory attention to complex sounds.

The dominance analysis also provides information about the relation between TRT and ESI. Both are measures of the ability to make use of partial information, but ESI is based on partial acoustic information about everyday sounds, and TRT involves the use of partial information about written words in sentences. That the two measures have very little common variance (0.5%) indicates that they are not measures of a common ability to use partial information to recreate wholes. Both measures have relatively high correlations with CogProc_Global, but ESI accounts for a greater proportion of variance in Speech Understanding independently of CogProc_Global (and the other measures) than does TRT. Thus, despite the common linguistic component in both TRT and the recognition of masked (or interrupted) speech (i.e., the use of linguistic context), the ability to identify familiar masked non-speech sounds is a better predictor of speech understanding in noise than is the ability to identify masked visually-presented words.

Measures from the test battery were less successful in accounting for individual differences in self-reported speech understanding difficulties, as measured by the SSQ. Only three of the predictors in Table 6 (HLoss_HarmMiss, CogProc_Global, and ESI) were significantly correlated with SSQ_Global. Together, these three variables accounted for 21.4% of the variance in SSQ_Global, or 33.4% (21.4/64) of the systematic variance. (Only an additional 7.8% can be accounted for by including the other six predictors in Table 6, with roughly 41% of that increase due to a suppressive effect of Age on HLoss_HarmMiss.) Dominance analysis was again used to examine the relative importance of the three significant predictors (see Table 8). Although these three measures were also important predictors of speech understanding, their relative importance is considerably different in this case. ESI is no longer dominant, accounting for considerably less of the explained variance than CogProc_Global, which is now completely dominant (accounting for the most variance at each level of *k*, and all comparisons within each level of *k*), and HLoss_HarmMiss, which has a much greater relative importance than it did in accounting for SpeechUnd_Global. The results suggest that while cognitive abilities and hearing loss are important predictors of both aided speech understanding and self-reported speech understanding difficulties, the latter is more influenced by variables not included in the current test battery.

**Table 8:** **Dominance analysis for prediction of SSQ_Global**.

***k***	**Cog_Global**	**ESI**	**HLoss_HM**
0	0.152	0.072	0.123
1	0.092	0.017	0.065
2	0.072	0.002	0.047
General dominance	0.105	0.031	0.078
Rescaled dominance	49.15	14.29	36.56

*R^2^-values associated with the addition of each variable to regression models consisting of subsets of other variables are presented as described in Table 7*.

## Summary

The main points of this study of individual differences in older adults follow. First, using the procedures and tasks in this study, it was possible to obtain reliable estimates of performance from older adults on many measures of non-speech auditory perception, visually based cognitive-linguistic processing, and speech understanding. Second, as a group, the older adults were outperformed by the group of young adults on about 25% of the measures used in this study. About half the time, however, these differences were in the cognitive domain and seldom were age-group differences observed in aided speech-understanding. The latter observation is undoubtedly due to the use of spectral shaping in this study to minimize the influence of stimulus inaudibility on speech-understanding performance. This suggests, however that neither the group differences in age nor presence of cochlear pathology were critical for recognizing or identifying the spectrally shaped speech stimuli. Third, individual differences in aided speech-understanding performance (SpeechUnd_Global) were well-explained by 5–6 predictor variables included in this study with significant contributions from visual measures of cognitive-linguistic processing (CogProc_Global, TRT), and non-speech auditory measures (ESI, Informational Masking, Hearing Loss and Dichotic Signal Detection) that primarily assess auditory abilities more complex than basic spectral and temporal processing. Fourth, self-report measures of speech-understanding difficulty (SSQ_Global), however, were less well-accounted for by the array of predictor variables included in this study. The three primary predictors that emerged were CogProc_Global, Hearing Loss, and, to a lesser degree, ESI, with about 33% of the systematic variance in SSQ scores explained. Given that the SSQ assesses auditory perception in many everyday listening situations and few of the subjects in this study were hearing aid wearers, it is to be expected, based on prior studies of unaided speech-understanding that hearing loss and cognition would be the primary predictors of individual differences (e.g., Akeroyd, 2008; Humes and Dubno, 2010).

### Conflict of interest statement

The authors declare that the research was conducted in the absence of any commercial or financial relationships that could be construed as a potential conflict of interest.
