# Corrigendum: Auditory and cognitive factors underlying individual differences in aided speech-understanding among older adults

**DOI:** 10.3389/fnsys.2016.00091

**Published:** 2016-11-11

**Authors:** Larry E. Humes, Gary R. Kidd, Jennifer J. Lentz

**Affiliations:** Department of Speech and Hearing Sciences, Indiana UniversityBloomington, IN, USA

**Keywords:** presbycusis, speech recognition, amplification, psychoacoustics, aging

Reason for Corrigendum:

There is an error in the reporting of the Text Recognition Threshold (TRT) data in the original article. The TRT results were reported as “percent unmasked” in Tables 1 and 2, but the data are actually percent *masked* values. Thus, higher scores mean better performance, and the older group actually performed slightly worse than the younger group on the TRT task (not better, as indicated in Table 2). The correlations with other measures, reported in Table 6, should be interpreted with the percent-masked scoring in mind. Thus, the significant positive correlations between TRT and the global measures of cognition and speech understanding reported in Table 6 indicate that better performance on the TRT is associated with higher (better) scores on those global measures.

## Conflict of interest statement

The authors declare that the research was conducted in the absence of any commercial or financial relationships that could be construed as a potential conflict of interest.

