# Supplementary material for: Auditory and cognitive factors underlying individual differences in aided speech-understanding among older adults
Source: Front Syst Neurosci. 2013 Oct 1;7:55. doi: 10.3389/fnsys.2013.00055 (PMC3787592; doi:10.3389/fnsys.2013.00055)
Supplement: Supplementary file 1 [file DataSheet1.PDF]

Correlations

Notes

Output Created		10-AUG-2013 11:02:13
Comments		
Input	Data	F: \TestBattery_DataAnalyses_2012\TestBattery_98OldOnly_Final_wEST_040913.sav
	Active Dataset	DataSet1
	Filter	<none>
	Weight	<none>
	Split File	<none>
	N of Rows in Working Data File	98
Missing Value Handling	Definition of Missing	User-defined missing values are treated as missing.
	Cases Used	Statistics for each pair of variables are based on all the cases with valid data for that pair.

Notes

Syntax		CORRELATIONS<br><br>/VARIABLES=infd_500_s_D_avg infd_500_d_D_avg infd_1000_s_D_avg infd_1000_d_D_avg mdetbb_20_avg mdi_5_dT_avg mdi_10_dT_avg mdi_10_iT_avg mdi_20_dT_avg mld_250_p_avg mld_500_p_avg iso_FF_avg hm_100_avg hm_200_avg str1_150_avg str2_150_avg str2_250_avg TBAC6avg ESTprcorrFNL MEMUPDATING SSPANMN SPATSHRTMEM AQTA1TIME AQTA2TIME AQTA3TIME TRTavg ISPINQUIET TCSPIN ISPINPL ISPINPH BSPINPL BSPINPH CRMND CRMSIMULTANEOUS CRMFo6STSEP CRMFo6STBACKWDS TOTSEQCORR SSQspeech SSQmod_spatial SSQmod_quality SSQmod_overall PTAtest hfPTAtest AGE<br>/PRINT=TWOTAIL SIG<br>/MISSING=PAIRWISE.
Resources	Processor Time	00:00:00.16
	Elapsed Time	00:00:00.12

Pearson r Correlations for all independent and dependent variables. Variables in same order as Table 2, plus hearing loss (PTA and HFPTA) and age added at the end.

Correlations

		infd_500_s_D_avg	infd_500_d_D_avg	infd_1000_s_D_avg
infd_500_s_D_avg	Pearson Correlation	1	.760	.774
	Sig. (2-tailed)		.000	.000
	N	98	98	98
infd_500_d_D_avg	Pearson Correlation	.760	1	.668
	Sig. (2-tailed)	.000		.000
	N	98	98	98
infd_1000_s_D_avg	Pearson Correlation	.774	.668	1
	Sig. (2-tailed)	.000	.000	
	N	98	98	98
infd_1000_d_D_avg	Pearson Correlation	.725	.870	.752
	Sig. (2-tailed)	.000	.000	.000
	N	98	98	98
mdetbb_20_avg	Pearson Correlation	.001	.017	.163
	Sig. (2-tailed)	.996	.869	.110
	N	97	97	97
mdi_5_dT_avg	Pearson Correlation	.147	.150	.231
	Sig. (2-tailed)	.151	.144	.023
	N	97	97	97
mdi_10_dT_avg	Pearson Correlation	.087	.157	.147
	Sig. (2-tailed)	.399	.124	.150
	N	97	97	97
mdi_10_iT_avg	Pearson Correlation	-.024	.052	.002
	Sig. (2-tailed)	.813	.611	.986
	N	97	97	97
mdi_20_dT_avg	Pearson Correlation	.127	.133	.159
	Sig. (2-tailed)	.217	.193	.120
	N	97	97	97
mld_250_p_avg	Pearson Correlation	.098	-.009	.132
	Sig. (2-tailed)	.351	.931	.208
	N	93	93	93
mld_500_p_avg	Pearson Correlation	.139	.078	.106
	Sig. (2-tailed)	.184	.457	.311
	N	93	93	93
iso_FF_avg	Pearson Correlation	.285	.274	.319
	Sig. (2-tailed)	.004	.006	.001
	N	98	98	98

Correlations

		infd_1000_d_D_avg	mdetbb_20_avg	mdi_5_dT_avg
infd_500_s_D_avg	Pearson Correlation	.725	.001	.147
	Sig. (2-tailed)	.000	.996	.151
	N	98	97	97
infd_500_d_D_avg	Pearson Correlation	.870	.017	.150
	Sig. (2-tailed)	.000	.869	.144
	N	98	97	97
infd_1000_s_D_avg	Pearson Correlation	.752	.163	.231
	Sig. (2-tailed)	.000	.110	.023
	N	98	97	97
infd_1000_d_D_avg	Pearson Correlation	1	.057	.140
	Sig. (2-tailed)		.579	.171
	N	98	97	97
mdetbb_20_avg	Pearson Correlation	.057	1	.266
	Sig. (2-tailed)	.579		.009
	N	97	97	96
mdi_5_dT_avg	Pearson Correlation	.140	.266	1
	Sig. (2-tailed)	.171	.009	
	N	97	96	97
mdi_10_dT_avg	Pearson Correlation	.149	.355	.693
	Sig. (2-tailed)	.144	.000	.000
	N	97	96	97
mdi_10_iT_avg	Pearson Correlation	-.024	.036	.093
	Sig. (2-tailed)	.813	.726	.366
	N	97	96	97
mdi_20_dT_avg	Pearson Correlation	.071	.318	.550
	Sig. (2-tailed)	.488	.002	.000
	N	97	96	97
mld_250_p_avg	Pearson Correlation	.118	.112	.289
	Sig. (2-tailed)	.262	.286	.005
	N	93	92	92
mld_500_p_avg	Pearson Correlation	.153	.019	.171
	Sig. (2-tailed)	.143	.860	.102
	N	93	92	92
iso_FF_avg	Pearson Correlation	.248	.219	.388
	Sig. (2-tailed)	.014	.031	.000
	N	98	97	97

Correlations

		mdi_10_dT_avg	mdi_10_iT_avg	mdi_20_dT_avg
infd_500_s_D_avg	Pearson Correlation	.087	-.024	.127
	Sig. (2-tailed)	.399	.813	.217
	N	97	97	97
infd_500_d_D_avg	Pearson Correlation	.157	.052	.133
	Sig. (2-tailed)	.124	.611	.193
	N	97	97	97
infd_1000_s_D_avg	Pearson Correlation	.147	.002	.159
	Sig. (2-tailed)	.150	.986	.120
	N	97	97	97
infd_1000_d_D_avg	Pearson Correlation	.149	-.024	.071
	Sig. (2-tailed)	.144	.813	.488
	N	97	97	97
mdetbb_20_avg	Pearson Correlation	.355	.036	.318
	Sig. (2-tailed)	.000	.726	.002
	N	96	96	96
mdi_5_dT_avg	Pearson Correlation	.693	.093	.550
	Sig. (2-tailed)	.000	.366	.000
	N	97	97	97
mdi_10_dT_avg	Pearson Correlation	1	.197	.791
	Sig. (2-tailed)		.053	.000
	N	97	97	97
mdi_10_iT_avg	Pearson Correlation	.197	1	.344
	Sig. (2-tailed)	.053		.001
	N	97	97	97
mdi_20_dT_avg	Pearson Correlation	.791	.344	1
	Sig. (2-tailed)	.000	.001	
	N	97	97	97
mld_250_p_avg	Pearson Correlation	.312	-.028	.256
	Sig. (2-tailed)	.002	.790	.014
	N	92	92	92
mld_500_p_avg	Pearson Correlation	.171	-.013	.105
	Sig. (2-tailed)	.104	.904	.318
	N	92	92	92
iso_FF_avg	Pearson Correlation	.406	.041	.379
	Sig. (2-tailed)	.000	.689	.000
	N	97	97	97

Correlations

		mld_250_p_avg	mld_500_p_avg	iso_FF_avg
infd_500_s_D_avg	Pearson Correlation	.098	.139	.285
	Sig. (2-tailed)	.351	.184	.004
	N	93	93	98
infd_500_d_D_avg	Pearson Correlation	-.009	.078	.274
	Sig. (2-tailed)	.931	.457	.006
	N	93	93	98
infd_1000_s_D_avg	Pearson Correlation	.132	.106	.319
	Sig. (2-tailed)	.208	.311	.001
	N	93	93	98
infd_1000_d_D_avg	Pearson Correlation	.118	.153	.248
	Sig. (2-tailed)	.262	.143	.014
	N	93	93	98
mdetbb_20_avg	Pearson Correlation	.112	.019	.219
	Sig. (2-tailed)	.286	.860	.031
	N	92	92	97
mdi_5_dT_avg	Pearson Correlation	.289	.171	.388
	Sig. (2-tailed)	.005	.102	.000
	N	92	92	97
mdi_10_dT_avg	Pearson Correlation	.312	.171	.406
	Sig. (2-tailed)	.002	.104	.000
	N	92	92	97
mdi_10_iT_avg	Pearson Correlation	-.028	-.013	.041
	Sig. (2-tailed)	.790	.904	.689
	N	92	92	97
mdi_20_dT_avg	Pearson Correlation	.256	.105	.379
	Sig. (2-tailed)	.014	.318	.000
	N	92	92	97
mld_250_p_avg	Pearson Correlation	1	.759	.163
	Sig. (2-tailed)		.000	.120
	N	93	93	93
mld_500_p_avg	Pearson Correlation	.759	1	.167
	Sig. (2-tailed)	.000		.110
	N	93	93	93
iso_FF_avg	Pearson Correlation	.163	.167	1
	Sig. (2-tailed)	.120	.110	
	N	93	93	98

Correlations

		hm_100_avg	hm_200_avg	str1_150_avg
infd_500_s_D_avg	Pearson Correlation	.364	.331	.258
	Sig. (2-tailed)	.000	.001	.010
	N	97	97	98
infd_500_d_D_avg	Pearson Correlation	.313	.346	.243
	Sig. (2-tailed)	.002	.001	.016
	N	97	97	98
infd_1000_s_D_avg	Pearson Correlation	.325	.260	.205
	Sig. (2-tailed)	.001	.010	.043
	N	97	97	98
infd_1000_d_D_avg	Pearson Correlation	.340	.336	.288
	Sig. (2-tailed)	.001	.001	.004
	N	97	97	98
mdetbb_20_avg	Pearson Correlation	.136	.045	.066
	Sig. (2-tailed)	.187	.667	.522
	N	96	96	97
mdi_5_dT_avg	Pearson Correlation	.216	.213	.024
	Sig. (2-tailed)	.035	.037	.815
	N	96	96	97
mdi_10_dT_avg	Pearson Correlation	.193	.223	-.103
	Sig. (2-tailed)	.060	.029	.317
	N	96	96	97
mdi_10_iT_avg	Pearson Correlation	-.081	-.106	-.125
	Sig. (2-tailed)	.435	.303	.221
	N	96	96	97
mdi_20_dT_avg	Pearson Correlation	.171	.214	-.125
	Sig. (2-tailed)	.095	.037	.222
	N	96	96	97
mld_250_p_avg	Pearson Correlation	.354	.192	-.144
	Sig. (2-tailed)	.001	.066	.169
	N	92	92	93
mld_500_p_avg	Pearson Correlation	.267	.248	-.134
	Sig. (2-tailed)	.010	.017	.200
	N	92	92	93
iso_FF_avg	Pearson Correlation	.227	.270	-.060
	Sig. (2-tailed)	.025	.007	.554
	N	97	97	98

Correlations

		str2_150_avg	str2_250_avg	TBAC6avg
infd_500_s_D_avg	Pearson Correlation	-.034	.019	-.322
	Sig. (2-tailed)	.736	.852	.001
	N	98	98	98
infd_500_d_D_avg	Pearson Correlation	.025	.062	-.274
	Sig. (2-tailed)	.811	.547	.006
	N	98	98	98
infd_1000_s_D_avg	Pearson Correlation	.064	.101	-.374
	Sig. (2-tailed)	.528	.320	.000
	N	98	98	98
infd_1000_d_D_avg	Pearson Correlation	.043	.090	-.309
	Sig. (2-tailed)	.675	.380	.002
	N	98	98	98
mdetbb_20_avg	Pearson Correlation	.031	.021	-.287
	Sig. (2-tailed)	.760	.841	.004
	N	97	97	97
mdi_5_dT_avg	Pearson Correlation	.176	.150	-.361
	Sig. (2-tailed)	.085	.142	.000
	N	97	97	97
mdi_10_dT_avg	Pearson Correlation	-.009	.000	-.270
	Sig. (2-tailed)	.931	.999	.007
	N	97	97	97
mdi_10_iT_avg	Pearson Correlation	-.187	-.132	.002
	Sig. (2-tailed)	.067	.198	.985
	N	97	97	97
mdi_20_dT_avg	Pearson Correlation	-.089	-.081	-.237
	Sig. (2-tailed)	.388	.428	.019
	N	97	97	97
mld_250_p_avg	Pearson Correlation	.076	.070	-.347
	Sig. (2-tailed)	.468	.506	.001
	N	93	93	93
mld_500_p_avg	Pearson Correlation	.055	.041	-.281
	Sig. (2-tailed)	.603	.696	.006
	N	93	93	93
iso_FF_avg	Pearson Correlation	-.040	-.024	-.395
	Sig. (2-tailed)	.692	.813	.000
	N	98	98	98

Correlations

		ESTpropcorr	Memory Updating:	Sentence Span SS Mean:
infd_500_s_D_avg	Pearson Correlation	-.143	-.181	-.239
	Sig. (2-tailed)	.160	.074	.018
	N	98	98	98
infd_500_d_D_avg	Pearson Correlation	-.105	-.132	-.229
	Sig. (2-tailed)	.305	.193	.024
	N	98	98	98
infd_1000_s_D_avg	Pearson Correlation	-.134	-.227	-.252
	Sig. (2-tailed)	.189	.025	.012
	N	98	98	98
infd_1000_d_D_avg	Pearson Correlation	-.155	-.181	-.184
	Sig. (2-tailed)	.127	.074	.069
	N	98	98	98
mdetbb_20_avg	Pearson Correlation	-.179	-.397	-.296
	Sig. (2-tailed)	.080	.000	.003
	N	97	97	97
mdi_5_dT_avg	Pearson Correlation	-.236	-.151	-.279
	Sig. (2-tailed)	.020	.139	.006
	N	97	97	97
mdi_10_dT_avg	Pearson Correlation	-.152	-.284	-.271
	Sig. (2-tailed)	.137	.005	.007
	N	97	97	97
mdi_10_iT_avg	Pearson Correlation	.086	-.055	-.047
	Sig. (2-tailed)	.402	.592	.649
	N	97	97	97
mdi_20_dT_avg	Pearson Correlation	-.144	-.291	-.259
	Sig. (2-tailed)	.158	.004	.010
	N	97	97	97
mld_250_p_avg	Pearson Correlation	-.332	-.301	-.312
	Sig. (2-tailed)	.001	.003	.002
	N	93	93	93
mld_500_p_avg	Pearson Correlation	-.345	-.290	-.291
	Sig. (2-tailed)	.001	.005	.005
	N	93	93	93
iso_FF_avg	Pearson Correlation	-.317	-.310	-.292
	Sig. (2-tailed)	.001	.002	.004
	N	98	98	98

Correlations

		Spatial Short Term Memory:	AQTA1TIME	AQTA2TIME
infd_500_s_D_avg	Pearson Correlation	-.083	.145	.152
	Sig. (2-tailed)	.419	.156	.134
	N	98	97	98
infd_500_d_D_avg	Pearson Correlation	-.110	.084	.100
	Sig. (2-tailed)	.280	.413	.327
	N	98	97	98
infd_1000_s_D_avg	Pearson Correlation	-.165	.123	.158
	Sig. (2-tailed)	.104	.229	.121
	N	98	97	98
infd_1000_d_D_avg	Pearson Correlation	-.169	.044	.078
	Sig. (2-tailed)	.097	.672	.443
	N	98	97	98
mdetbb_20_avg	Pearson Correlation	-.185	.210	.179
	Sig. (2-tailed)	.069	.040	.079
	N	97	96	97
mdi_5_dT_avg	Pearson Correlation	-.148	.203	.177
	Sig. (2-tailed)	.147	.048	.084
	N	97	96	97
mdi_10_dT_avg	Pearson Correlation	-.223	.134	.048
	Sig. (2-tailed)	.028	.194	.639
	N	97	96	97
mdi_10_iT_avg	Pearson Correlation	-.082	-.103	-.060
	Sig. (2-tailed)	.425	.316	.557
	N	97	96	97
mdi_20_dT_avg	Pearson Correlation	-.217	.146	.058
	Sig. (2-tailed)	.032	.156	.569
	N	97	96	97
mld_250_p_avg	Pearson Correlation	-.228	.218	.151
	Sig. (2-tailed)	.028	.037	.149
	N	93	92	93
mld_500_p_avg	Pearson Correlation	-.211	.300	.234
	Sig. (2-tailed)	.042	.004	.024
	N	93	92	93
iso_FF_avg	Pearson Correlation	-.161	.239	.313
	Sig. (2-tailed)	.114	.018	.002
	N	98	97	98

Correlations

		AQTA3TIME	TRTavg	iSPIN_Q_Overall
infd_500_s_D_avg	Pearson Correlation	.142	-.100	-.163
	Sig. (2-tailed)	.166	.328	.119
	N	97	98	93
infd_500_d_D_avg	Pearson Correlation	.114	-.147	-.181
	Sig. (2-tailed)	.265	.147	.082
	N	97	98	93
infd_1000_s_D_avg	Pearson Correlation	.098	-.147	-.161
	Sig. (2-tailed)	.339	.150	.123
	N	97	98	93
infd_1000_d_D_avg	Pearson Correlation	.085	-.148	-.198
	Sig. (2-tailed)	.409	.147	.057
	N	97	98	93
mdetbb_20_avg	Pearson Correlation	.266	-.125	-.159
	Sig. (2-tailed)	.009	.222	.130
	N	96	97	92
mdi_5_dT_avg	Pearson Correlation	.131	-.128	-.012
	Sig. (2-tailed)	.203	.210	.913
	N	96	97	92
mdi_10_dT_avg	Pearson Correlation	.109	-.235	.013
	Sig. (2-tailed)	.292	.021	.903
	N	96	97	92
mdi_10_iT_avg	Pearson Correlation	-.059	-.055	.066
	Sig. (2-tailed)	.570	.594	.531
	N	96	97	92
mdi_20_dT_avg	Pearson Correlation	.101	-.215	-.014
	Sig. (2-tailed)	.327	.034	.892
	N	96	97	92
mld_250_p_avg	Pearson Correlation	.239	-.424	-.327
	Sig. (2-tailed)	.022	.000	.002
	N	92	93	88
mld_500_p_avg	Pearson Correlation	.354	-.530	-.362
	Sig. (2-tailed)	.001	.000	.001
	N	92	93	88
iso_FF_avg	Pearson Correlation	.282	-.120	-.080
	Sig. (2-tailed)	.005	.238	.447
	N	97	98	93

Correlations

		Time-compressed SPIN:	ISPIN PL MEAN:	ISPIN PH MEAN:
infd_500_s_D_avg	Pearson Correlation	-.265	-.218	-.272
	Sig. (2-tailed)	.008	.031	.007
	N	98	98	98
infd_500_d_D_avg	Pearson Correlation	-.252	-.134	-.233
	Sig. (2-tailed)	.012	.187	.021
	N	98	98	98
infd_1000_s_D_avg	Pearson Correlation	-.238	-.250	-.236
	Sig. (2-tailed)	.018	.013	.019
	N	98	98	98
infd_1000_d_D_avg	Pearson Correlation	-.297	-.246	-.280
	Sig. (2-tailed)	.003	.014	.005
	N	98	98	98
mdetbb_20_avg	Pearson Correlation	-.120	-.173	-.221
	Sig. (2-tailed)	.242	.090	.030
	N	97	97	97
mdi_5_dT_avg	Pearson Correlation	-.167	-.135	-.084
	Sig. (2-tailed)	.101	.188	.412
	N	97	97	97
mdi_10_dT_avg	Pearson Correlation	-.062	-.121	-.171
	Sig. (2-tailed)	.549	.236	.094
	N	97	97	97
mdi_10_iT_avg	Pearson Correlation	.220	.108	.120
	Sig. (2-tailed)	.031	.293	.242
	N	97	97	97
mdi_20_dT_avg	Pearson Correlation	-.016	-.115	-.130
	Sig. (2-tailed)	.875	.262	.205
	N	97	97	97
mld_250_p_avg	Pearson Correlation	-.387	-.367	-.381
	Sig. (2-tailed)	.000	.000	.000
	N	93	93	93
mld_500_p_avg	Pearson Correlation	-.450	-.450	-.391
	Sig. (2-tailed)	.000	.000	.000
	N	93	93	93
iso_FF_avg	Pearson Correlation	-.170	-.181	-.212
	Sig. (2-tailed)	.094	.075	.036
	N	98	98	98

Correlations

		BSPIN PL MEAN:	BSPIN PH MEAN:	No distractor:
infd_500_s_D_avg	Pearson Correlation	-.171	-.157	-.215
	Sig. (2-tailed)	.093	.122	.034
	N	98	98	98
infd_500_d_D_avg	Pearson Correlation	-.154	-.175	-.204
	Sig. (2-tailed)	.129	.084	.044
	N	98	98	98
infd_1000_s_D_avg	Pearson Correlation	-.147	-.149	-.123
	Sig. (2-tailed)	.148	.142	.226
	N	98	98	98
infd_1000_d_D_avg	Pearson Correlation	-.209	-.169	-.194
	Sig. (2-tailed)	.039	.096	.056
	N	98	98	98
mdetbb_20_avg	Pearson Correlation	-.072	-.108	-.133
	Sig. (2-tailed)	.485	.291	.193
	N	97	97	97
mdi_5_dT_avg	Pearson Correlation	-.134	-.025	-.032
	Sig. (2-tailed)	.190	.811	.759
	N	97	97	97
mdi_10_dT_avg	Pearson Correlation	-.068	-.028	-.075
	Sig. (2-tailed)	.506	.787	.462
	N	97	97	97
mdi_10_iT_avg	Pearson Correlation	.183	.074	.080
	Sig. (2-tailed)	.072	.473	.438
	N	97	97	97
mdi_20_dT_avg	Pearson Correlation	-.079	-.055	-.019
	Sig. (2-tailed)	.442	.591	.852
	N	97	97	97
mld_250_p_avg	Pearson Correlation	-.358	-.294	-.232
	Sig. (2-tailed)	.000	.004	.025
	N	93	93	93
mld_500_p_avg	Pearson Correlation	-.447	-.248	-.201
	Sig. (2-tailed)	.000	.017	.054
	N	93	93	93
iso_FF_avg	Pearson Correlation	-.156	-.039	-.062
	Sig. (2-tailed)	.124	.704	.545
	N	98	98	98

Correlations

		Simultaneous:	Fo 6ST separation:	Backwards 6ST distract:
infd_500_s_D_avg	Pearson Correlation	-.283	-.402	-.294
	Sig. (2-tailed)	.005	.000	.003
	N	98	98	98
infd_500_d_D_avg	Pearson Correlation	-.265	-.320	-.245
	Sig. (2-tailed)	.008	.001	.015
	N	98	98	98
infd_1000_s_D_avg	Pearson Correlation	-.215	-.325	-.165
	Sig. (2-tailed)	.033	.001	.104
	N	98	98	98
infd_1000_d_D_avg	Pearson Correlation	-.296	-.344	-.257
	Sig. (2-tailed)	.003	.001	.010
	N	98	98	98
mdetbb_20_avg	Pearson Correlation	-.120	-.247	-.116
	Sig. (2-tailed)	.243	.015	.259
	N	97	97	97
mdi_5_dT_avg	Pearson Correlation	-.033	-.164	-.058
	Sig. (2-tailed)	.751	.109	.571
	N	97	97	97
mdi_10_dT_avg	Pearson Correlation	.026	-.220	-.112
	Sig. (2-tailed)	.803	.031	.276
	N	97	97	97
mdi_10_iT_avg	Pearson Correlation	-.016	.004	.072
	Sig. (2-tailed)	.880	.969	.485
	N	97	97	97
mdi_20_dT_avg	Pearson Correlation	-.039	-.209	-.098
	Sig. (2-tailed)	.705	.040	.339
	N	97	97	97
mld_250_p_avg	Pearson Correlation	-.170	-.332	-.259
	Sig. (2-tailed)	.103	.001	.012
	N	93	93	93
mld_500_p_avg	Pearson Correlation	-.149	-.374	-.222
	Sig. (2-tailed)	.153	.000	.033
	N	93	93	93
iso_FF_avg	Pearson Correlation	-.178	-.141	-.033
	Sig. (2-tailed)	.079	.168	.746
	N	98	98	98

Correlations

		TOTSEQCORR	SSQspeech	SSQmod_spatial
infd_500_s_D_avg	Pearson Correlation	-.380	-.082	.040
	Sig. (2-tailed)	.000	.420	.699
	N	95	98	98
infd_500_d_D_avg	Pearson Correlation	-.193	-.011	.057
	Sig. (2-tailed)	.061	.918	.580
	N	95	98	98
infd_1000_s_D_avg	Pearson Correlation	-.403	.049	.016
	Sig. (2-tailed)	.000	.633	.873
	N	95	98	98
infd_1000_d_D_avg	Pearson Correlation	-.294	.030	.041
	Sig. (2-tailed)	.004	.772	.690
	N	95	98	98
mdetbb_20_avg	Pearson Correlation	-.256	-.077	-.206
	Sig. (2-tailed)	.013	.451	.043
	N	94	97	97
mdi_5_dT_avg	Pearson Correlation	-.227	-.160	-.218
	Sig. (2-tailed)	.028	.117	.032
	N	94	97	97
mdi_10_dT_avg	Pearson Correlation	-.185	-.160	-.197
	Sig. (2-tailed)	.074	.116	.053
	N	94	97	97
mdi_10_iT_avg	Pearson Correlation	.053	.234	-.015
	Sig. (2-tailed)	.609	.021	.883
	N	94	97	97
mdi_20_dT_avg	Pearson Correlation	-.220	-.014	-.191
	Sig. (2-tailed)	.033	.893	.060
	N	94	97	97
mld_250_p_avg	Pearson Correlation	-.294	-.081	-.217
	Sig. (2-tailed)	.005	.440	.036
	N	91	93	93
mld_500_p_avg	Pearson Correlation	-.290	-.120	-.203
	Sig. (2-tailed)	.005	.253	.051
	N	91	93	93
iso_FF_avg	Pearson Correlation	-.237	-.098	-.202
	Sig. (2-tailed)	.021	.338	.046
	N	95	98	98

Correlations

		SSQmod_quality	SSQmod_overall	PTAtest	hfPTAtest
infd_500_s_D_avg	Pearson Correlation	-.044	-.033	.088	.042
	Sig. (2-tailed)	.667	.744	.387	.683
	N	97	98	98	98
infd_500_d_D_avg	Pearson Correlation	.020	.026	.094	.063
	Sig. (2-tailed)	.847	.803	.358	.539
	N	97	98	98	98
infd_1000_s_D_avg	Pearson Correlation	-.066	.006	.058	-.055
	Sig. (2-tailed)	.519	.953	.573	.588
	N	97	98	98	98
infd_1000_d_D_avg	Pearson Correlation	-.008	.028	.091	.022
	Sig. (2-tailed)	.938	.786	.375	.833
	N	97	98	98	98
mdetbb_20_avg	Pearson Correlation	-.172	-.179	.014	-.108
	Sig. (2-tailed)	.094	.079	.890	.291
	N	96	97	97	97
mdi_5_dT_avg	Pearson Correlation	-.283	-.247	.097	.090
	Sig. (2-tailed)	.005	.015	.345	.378
	N	96	97	97	97
mdi_10_dT_avg	Pearson Correlation	-.204	-.214	-.005	-.044
	Sig. (2-tailed)	.046	.035	.960	.669
	N	96	97	97	97
mdi_10_iT_avg	Pearson Correlation	.080	.111	-.248	-.240
	Sig. (2-tailed)	.440	.278	.014	.018
	N	96	97	97	97
mdi_20_dT_avg	Pearson Correlation	-.165	-.136	-.048	-.052
	Sig. (2-tailed)	.109	.184	.638	.610
	N	96	97	97	97
mld_250_p_avg	Pearson Correlation	-.270	-.226	.471	.378
	Sig. (2-tailed)	.009	.029	.000	.000
	N	92	93	93	93
mld_500_p_avg	Pearson Correlation	-.263	-.236	.450	.422
	Sig. (2-tailed)	.011	.023	.000	.000
	N	92	93	93	93
iso_FF_avg	Pearson Correlation	-.283	-.213	.190	.062
	Sig. (2-tailed)	.005	.035	.061	.544
	N	97	98	98	98

Correlations

		AGE
infd_500_s_D_avg	Pearson Correlation	.114
	Sig. (2-tailed)	.262
	N	98
infd_500_d_D_avg	Pearson Correlation	.133
	Sig. (2-tailed)	.190
	N	98
infd_1000_s_D_avg	Pearson Correlation	.063
	Sig. (2-tailed)	.539
	N	98
infd_1000_d_D_avg	Pearson Correlation	.195
	Sig. (2-tailed)	.055
	N	98
mdetbb_20_avg	Pearson Correlation	.008
	Sig. (2-tailed)	.940
	N	97
mdi_5_dT_avg	Pearson Correlation	-.070
	Sig. (2-tailed)	.495
	N	97
mdi_10_dT_avg	Pearson Correlation	-.099
	Sig. (2-tailed)	.336
	N	97
mdi_10_iT_avg	Pearson Correlation	-.160
	Sig. (2-tailed)	.117
	N	97
mdi_20_dT_avg	Pearson Correlation	-.083
	Sig. (2-tailed)	.420
	N	97
mld_250_p_avg	Pearson Correlation	.275
	Sig. (2-tailed)	.008
	N	93
mld_500_p_avg	Pearson Correlation	.349
	Sig. (2-tailed)	.001
	N	93
iso_FF_avg	Pearson Correlation	-.004
	Sig. (2-tailed)	.971
	N	98

Correlations

		infd_500_s_D_avg	infd_500_d_D_avg	infd_1000_s_D_avg
hm_100_avg	Pearson Correlation	.364	.313	.325
	Sig. (2-tailed)	.000	.002	.001
	N	97	97	97
hm_200_avg	Pearson Correlation	.331	.346	.260
	Sig. (2-tailed)	.001	.001	.010
	N	97	97	97
str1_150_avg	Pearson Correlation	.258	.243	.205
	Sig. (2-tailed)	.010	.016	.043
	N	98	98	98
str2_150_avg	Pearson Correlation	-.034	.025	.064
	Sig. (2-tailed)	.736	.811	.528
	N	98	98	98
str2_250_avg	Pearson Correlation	.019	.062	.101
	Sig. (2-tailed)	.852	.547	.320
	N	98	98	98
TBAC6avg	Pearson Correlation	-.322	-.274	-.374
	Sig. (2-tailed)	.001	.006	.000
	N	98	98	98
ESTpropcorr	Pearson Correlation	-.143	-.105	-.134
	Sig. (2-tailed)	.160	.305	.189
	N	98	98	98
Memory Updating:	Pearson Correlation	-.181	-.132	-.227
	Sig. (2-tailed)	.074	.193	.025
	N	98	98	98
Sentence Span SS Mean:	Pearson Correlation	-.239	-.229	-.252
	Sig. (2-tailed)	.018	.024	.012
	N	98	98	98
Spatial Short Term Memory:	Pearson Correlation	-.083	-.110	-.165
	Sig. (2-tailed)	.419	.280	.104
	N	98	98	98
AQTA1TIME	Pearson Correlation	.145	.084	.123
	Sig. (2-tailed)	.156	.413	.229
	N	97	97	97
AQTA2TIME	Pearson Correlation	.152	.100	.158
	Sig. (2-tailed)	.134	.327	.121
	N	98	98	98

Correlations

		infd_1000_d_D_avg	mdetbb_20_avg	mdi_5_dT_avg
hm_100_avg	Pearson Correlation	.340	.136	.216
	Sig. (2-tailed)	.001	.187	.035
	N	97	96	96
hm_200_avg	Pearson Correlation	.336	.045	.213
	Sig. (2-tailed)	.001	.667	.037
	N	97	96	96
str1_150_avg	Pearson Correlation	.288	.066	.024
	Sig. (2-tailed)	.004	.522	.815
	N	98	97	97
str2_150_avg	Pearson Correlation	.043	.031	.176
	Sig. (2-tailed)	.675	.760	.085
	N	98	97	97
str2_250_avg	Pearson Correlation	.090	.021	.150
	Sig. (2-tailed)	.380	.841	.142
	N	98	97	97
TBAC6avg	Pearson Correlation	-.309	-.287	-.361
	Sig. (2-tailed)	.002	.004	.000
	N	98	97	97
ESTpropcorr	Pearson Correlation	-.155	-.179	-.236
	Sig. (2-tailed)	.127	.080	.020
	N	98	97	97
Memory Updating:	Pearson Correlation	-.181	-.397	-.151
	Sig. (2-tailed)	.074	.000	.139
	N	98	97	97
Sentence Span SS Mean:	Pearson Correlation	-.184	-.296	-.279
	Sig. (2-tailed)	.069	.003	.006
	N	98	97	97
Spatial Short Term Memory:	Pearson Correlation	-.169	-.185	-.148
	Sig. (2-tailed)	.097	.069	.147
	N	98	97	97
AQTA1TIME	Pearson Correlation	.044	.210	.203
	Sig. (2-tailed)	.672	.040	.048
	N	97	96	96
AQTA2TIME	Pearson Correlation	.078	.179	.177
	Sig. (2-tailed)	.443	.079	.084
	N	98	97	97

Correlations

		mdi_10_dT_avg	mdi_10_iT_avg	mdi_20_dT_avg
hm_100_avg	Pearson Correlation	.193	-.081	.171
	Sig. (2-tailed)	.060	.435	.095
	N	96	96	96
hm_200_avg	Pearson Correlation	.223	-.106	.214
	Sig. (2-tailed)	.029	.303	.037
	N	96	96	96
str1_150_avg	Pearson Correlation	-.103	-.125	-.125
	Sig. (2-tailed)	.317	.221	.222
	N	97	97	97
str2_150_avg	Pearson Correlation	-.009	-.187	-.089
	Sig. (2-tailed)	.931	.067	.388
	N	97	97	97
str2_250_avg	Pearson Correlation	.000	-.132	-.081
	Sig. (2-tailed)	.999	.198	.428
	N	97	97	97
TBAC6avg	Pearson Correlation	-.270	.002	-.237
	Sig. (2-tailed)	.007	.985	.019
	N	97	97	97
ESTpropcorr	Pearson Correlation	-.152	.086	-.144
	Sig. (2-tailed)	.137	.402	.158
	N	97	97	97
Memory Updating:	Pearson Correlation	-.284	-.055	-.291
	Sig. (2-tailed)	.005	.592	.004
	N	97	97	97
Sentence Span SS Mean:	Pearson Correlation	-.271	-.047	-.259
	Sig. (2-tailed)	.007	.649	.010
	N	97	97	97
Spatial Short Term Memory:	Pearson Correlation	-.223	-.082	-.217
	Sig. (2-tailed)	.028	.425	.032
	N	97	97	97
AQTA1TIME	Pearson Correlation	.134	-.103	.146
	Sig. (2-tailed)	.194	.316	.156
	N	96	96	96
AQTA2TIME	Pearson Correlation	.048	-.060	.058
	Sig. (2-tailed)	.639	.557	.569
	N	97	97	97

Correlations

		mld_250_p_avg	mld_500_p_avg	iso_FF_avg
hm_100_avg	Pearson Correlation	.354	.267	.227
	Sig. (2-tailed)	.001	.010	.025
	N	92	92	97
hm_200_avg	Pearson Correlation	.192	.248	.270
	Sig. (2-tailed)	.066	.017	.007
	N	92	92	97
str1_150_avg	Pearson Correlation	-.144	-.134	-.060
	Sig. (2-tailed)	.169	.200	.554
	N	93	93	98
str2_150_avg	Pearson Correlation	.076	.055	-.040
	Sig. (2-tailed)	.468	.603	.692
	N	93	93	98
str2_250_avg	Pearson Correlation	.070	.041	-.024
	Sig. (2-tailed)	.506	.696	.813
	N	93	93	98
TBAC6avg	Pearson Correlation	-.347	-.281	-.395
	Sig. (2-tailed)	.001	.006	.000
	N	93	93	98
ESTpropcorr	Pearson Correlation	-.332	-.345	-.317
	Sig. (2-tailed)	.001	.001	.001
	N	93	93	98
Memory Updating:	Pearson Correlation	-.301	-.290	-.310
	Sig. (2-tailed)	.003	.005	.002
	N	93	93	98
Sentence Span SS Mean:	Pearson Correlation	-.312	-.291	-.292
	Sig. (2-tailed)	.002	.005	.004
	N	93	93	98
Spatial Short Term Memory:	Pearson Correlation	-.228	-.211	-.161
	Sig. (2-tailed)	.028	.042	.114
	N	93	93	98
AQTA1TIME	Pearson Correlation	.218	.300	.239
	Sig. (2-tailed)	.037	.004	.018
	N	92	92	97
AQTA2TIME	Pearson Correlation	.151	.234	.313
	Sig. (2-tailed)	.149	.024	.002
	N	93	93	98

Correlations

		hm_100_avg	hm_200_avg	str1_150_avg
hm_100_avg	Pearson Correlation	1	.581	.042
	Sig. (2-tailed)		.000	.685
	N	97	97	97
hm_200_avg	Pearson Correlation	.581	1	.139
	Sig. (2-tailed)	.000		.175
	N	97	97	97
str1_150_avg	Pearson Correlation	.042	.139	1
	Sig. (2-tailed)	.685	.175	
	N	97	97	98
str2_150_avg	Pearson Correlation	.021	-.006	.278
	Sig. (2-tailed)	.835	.956	.006
	N	97	97	98
str2_250_avg	Pearson Correlation	.045	.024	.318
	Sig. (2-tailed)	.662	.818	.001
	N	97	97	98
TBAC6avg	Pearson Correlation	-.293	-.304	.052
	Sig. (2-tailed)	.004	.002	.612
	N	97	97	98
ESTpropcorr	Pearson Correlation	-.220	-.247	.098
	Sig. (2-tailed)	.030	.015	.338
	N	97	97	98
Memory Updating:	Pearson Correlation	-.223	-.210	.123
	Sig. (2-tailed)	.028	.039	.227
	N	97	97	98
Sentence Span SS Mean:	Pearson Correlation	-.198	-.385	.056
	Sig. (2-tailed)	.052	.000	.585
	N	97	97	98
Spatial Short Term Memory:	Pearson Correlation	-.165	-.230	.142
	Sig. (2-tailed)	.107	.024	.162
	N	97	97	98
AQTA1TIME	Pearson Correlation	.280	.347	.113
	Sig. (2-tailed)	.006	.001	.269
	N	96	96	97
AQTA2TIME	Pearson Correlation	.164	.203	.015
	Sig. (2-tailed)	.108	.047	.887
	N	97	97	98

Correlations

		str2_150_avg	str2_250_avg	TBAC6avg
hm_100_avg	Pearson Correlation	.021	.045	-.293
	Sig. (2-tailed)	.835	.662	.004
	N	97	97	97
hm_200_avg	Pearson Correlation	-.006	.024	-.304
	Sig. (2-tailed)	.956	.818	.002
	N	97	97	97
str1_150_avg	Pearson Correlation	.278	.318	.052
	Sig. (2-tailed)	.006	.001	.612
	N	98	98	98
str2_150_avg	Pearson Correlation	1	.944	-.029
	Sig. (2-tailed)		.000	.777
	N	98	98	98
str2_250_avg	Pearson Correlation	.944	1	-.012
	Sig. (2-tailed)	.000		.903
	N	98	98	98
TBAC6avg	Pearson Correlation	-.029	-.012	1
	Sig. (2-tailed)	.777	.903	
	N	98	98	98
ESTpropcorr	Pearson Correlation	-.232	-.195	.200
	Sig. (2-tailed)	.022	.054	.048
	N	98	98	98
Memory Updating:	Pearson Correlation	.003	.036	.412
	Sig. (2-tailed)	.980	.725	.000
	N	98	98	98
Sentence Span SS Mean:	Pearson Correlation	-.105	-.091	.586
	Sig. (2-tailed)	.305	.370	.000
	N	98	98	98
Spatial Short Term Memory:	Pearson Correlation	-.110	-.060	.257
	Sig. (2-tailed)	.281	.555	.011
	N	98	98	98
AQTA1TIME	Pearson Correlation	.285	.282	-.310
	Sig. (2-tailed)	.005	.005	.002
	N	97	97	97
AQTA2TIME	Pearson Correlation	.137	.129	-.396
	Sig. (2-tailed)	.180	.204	.000
	N	98	98	98

Correlations

		ESTpropcorr	Memory Updating:	Sentence Span SS Mean:
hm_100_avg	Pearson Correlation	-.220	-.223	-.198
	Sig. (2-tailed)	.030	.028	.052
	N	97	97	97
hm_200_avg	Pearson Correlation	-.247	-.210	-.385
	Sig. (2-tailed)	.015	.039	.000
	N	97	97	97
str1_150_avg	Pearson Correlation	.098	.123	.056
	Sig. (2-tailed)	.338	.227	.585
	N	98	98	98
str2_150_avg	Pearson Correlation	-.232	.003	-.105
	Sig. (2-tailed)	.022	.980	.305
	N	98	98	98
str2_250_avg	Pearson Correlation	-.195	.036	-.091
	Sig. (2-tailed)	.054	.725	.370
	N	98	98	98
TBAC6avg	Pearson Correlation	.200	.412	.586
	Sig. (2-tailed)	.048	.000	.000
	N	98	98	98
ESTpropcorr	Pearson Correlation	1	.323	.315
	Sig. (2-tailed)		.001	.002
	N	98	98	98
Memory Updating:	Pearson Correlation	.323	1	.628
	Sig. (2-tailed)	.001		.000
	N	98	98	98
Sentence Span SS Mean:	Pearson Correlation	.315	.628	1
	Sig. (2-tailed)	.002	.000	
	N	98	98	98
Spatial Short Term Memory:	Pearson Correlation	.335	.539	.341
	Sig. (2-tailed)	.001	.000	.001
	N	98	98	98
AQTA1TIME	Pearson Correlation	-.215	-.358	-.420
	Sig. (2-tailed)	.035	.000	.000
	N	97	97	97
AQTA2TIME	Pearson Correlation	-.256	-.473	-.492
	Sig. (2-tailed)	.011	.000	.000
	N	98	98	98

Correlations

		Spatial Short Term Memory:	AQTA1TIME	AQTA2TIME
hm_100_avg	Pearson Correlation	-.165	.280	.164
	Sig. (2-tailed)	.107	.006	.108
	N	97	96	97
hm_200_avg	Pearson Correlation	-.230	.347	.203
	Sig. (2-tailed)	.024	.001	.047
	N	97	96	97
str1_150_avg	Pearson Correlation	.142	.113	.015
	Sig. (2-tailed)	.162	.269	.887
	N	98	97	98
str2_150_avg	Pearson Correlation	-.110	.285	.137
	Sig. (2-tailed)	.281	.005	.180
	N	98	97	98
str2_250_avg	Pearson Correlation	-.060	.282	.129
	Sig. (2-tailed)	.555	.005	.204
	N	98	97	98
TBAC6avg	Pearson Correlation	.257	-.310	-.396
	Sig. (2-tailed)	.011	.002	.000
	N	98	97	98
ESTpropcorr	Pearson Correlation	.335	-.215	-.256
	Sig. (2-tailed)	.001	.035	.011
	N	98	97	98
Memory Updating:	Pearson Correlation	.539	-.358	-.473
	Sig. (2-tailed)	.000	.000	.000
	N	98	97	98
Sentence Span SS Mean:	Pearson Correlation	.341	-.420	-.492
	Sig. (2-tailed)	.001	.000	.000
	N	98	97	98
Spatial Short Term Memory:	Pearson Correlation	1	-.187	-.158
	Sig. (2-tailed)		.066	.120
	N	98	97	98
AQTA1TIME	Pearson Correlation	-.187	1	.735
	Sig. (2-tailed)	.066		.000
	N	97	97	97
AQTA2TIME	Pearson Correlation	-.158	.735	1
	Sig. (2-tailed)	.120	.000	
	N	98	97	98

Correlations

		AQTA3TIME	TRTavg	iSPIN_Q_Overall
hm_100_avg	Pearson Correlation	.179	-.271	-.208
	Sig. (2-tailed)	.081	.007	.047
	N	96	97	92
hm_200_avg	Pearson Correlation	.186	-.446	-.187
	Sig. (2-tailed)	.069	.000	.075
	N	96	97	92
str1_150_avg	Pearson Correlation	-.061	.127	.170
	Sig. (2-tailed)	.553	.211	.104
	N	97	98	93
str2_150_avg	Pearson Correlation	.115	.039	.023
	Sig. (2-tailed)	.260	.703	.826
	N	97	98	93
str2_250_avg	Pearson Correlation	.115	.018	.009
	Sig. (2-tailed)	.262	.857	.932
	N	97	98	93
TBAC6avg	Pearson Correlation	-.354	.401	.495
	Sig. (2-tailed)	.000	.000	.000
	N	97	98	93
ESTpropcorr	Pearson Correlation	-.292	.073	.250
	Sig. (2-tailed)	.004	.475	.016
	N	97	98	93
Memory Updating:	Pearson Correlation	-.471	.394	.353
	Sig. (2-tailed)	.000	.000	.001
	N	97	98	93
Sentence Span SS Mean:	Pearson Correlation	-.481	.539	.514
	Sig. (2-tailed)	.000	.000	.000
	N	97	98	93
Spatial Short Term Memory:	Pearson Correlation	-.194	.216	.145
	Sig. (2-tailed)	.056	.033	.165
	N	97	98	93
AQTA1TIME	Pearson Correlation	.716	-.422	-.144
	Sig. (2-tailed)	.000	.000	.171
	N	97	97	92
AQTA2TIME	Pearson Correlation	.771	-.358	-.443
	Sig. (2-tailed)	.000	.000	.000
	N	97	98	93

Correlations

		Time-compressed SPIN:	ISPIN PL MEAN:	ISPIN PH MEAN:
hm_100_avg	Pearson Correlation	-.387	-.387	-.413
	Sig. (2-tailed)	.000	.000	.000
	N	97	97	97
hm_200_avg	Pearson Correlation	-.420	-.398	-.454
	Sig. (2-tailed)	.000	.000	.000
	N	97	97	97
str1_150_avg	Pearson Correlation	.080	.083	.137
	Sig. (2-tailed)	.434	.414	.178
	N	98	98	98
str2_150_avg	Pearson Correlation	-.208	.009	-.049
	Sig. (2-tailed)	.040	.932	.629
	N	98	98	98
str2_250_avg	Pearson Correlation	-.211	.002	-.071
	Sig. (2-tailed)	.037	.984	.487
	N	98	98	98
TBAC6avg	Pearson Correlation	.448	.258	.377
	Sig. (2-tailed)	.000	.010	.000
	N	98	98	98
ESTpropcorr	Pearson Correlation	.485	.449	.537
	Sig. (2-tailed)	.000	.000	.000
	N	98	98	98
Memory Updating:	Pearson Correlation	.337	.372	.360
	Sig. (2-tailed)	.001	.000	.000
	N	98	98	98
Sentence Span SS Mean:	Pearson Correlation	.484	.380	.470
	Sig. (2-tailed)	.000	.000	.000
	N	98	98	98
Spatial Short Term Memory:	Pearson Correlation	.157	.263	.156
	Sig. (2-tailed)	.122	.009	.125
	N	98	98	98
AQTA1TIME	Pearson Correlation	-.342	-.278	-.288
	Sig. (2-tailed)	.001	.006	.004
	N	97	97	97
AQTA2TIME	Pearson Correlation	-.476	-.339	-.363
	Sig. (2-tailed)	.000	.001	.000
	N	98	98	98

Correlations

		BSPIN PL MEAN:	BSPIN PH MEAN:	No distractor:
hm_100_avg	Pearson Correlation	-.329	-.219	-.364
	Sig. (2-tailed)	.001	.031	.000
	N	97	97	97
hm_200_avg	Pearson Correlation	-.410	-.243	-.363
	Sig. (2-tailed)	.000	.017	.000
	N	97	97	97
str1_150_avg	Pearson Correlation	.142	.209	.028
	Sig. (2-tailed)	.164	.039	.781
	N	98	98	98
str2_150_avg	Pearson Correlation	-.191	-.087	-.149
	Sig. (2-tailed)	.060	.394	.142
	N	98	98	98
str2_250_avg	Pearson Correlation	-.157	-.096	-.132
	Sig. (2-tailed)	.122	.348	.194
	N	98	98	98
TBAC6avg	Pearson Correlation	.268	.451	.305
	Sig. (2-tailed)	.008	.000	.002
	N	98	98	98
ESTpropcorr	Pearson Correlation	.537	.317	.312
	Sig. (2-tailed)	.000	.001	.002
	N	98	98	98
Memory Updating:	Pearson Correlation	.358	.302	.239
	Sig. (2-tailed)	.000	.002	.018
	N	98	98	98
Sentence Span SS Mean:	Pearson Correlation	.430	.542	.462
	Sig. (2-tailed)	.000	.000	.000
	N	98	98	98
Spatial Short Term Memory:	Pearson Correlation	.193	.046	.166
	Sig. (2-tailed)	.057	.656	.102
	N	98	98	98
AQTA1TIME	Pearson Correlation	-.367	-.150	-.164
	Sig. (2-tailed)	.000	.142	.108
	N	97	97	97
AQTA2TIME	Pearson Correlation	-.484	-.388	-.246
	Sig. (2-tailed)	.000	.000	.015
	N	98	98	98

Correlations

		Simultaneous:	Fo 6ST separation:	Backwards 6ST distract:
hm_100_avg	Pearson Correlation	-.124	-.398	-.395
	Sig. (2-tailed)	.226	.000	.000
	N	97	97	97
hm_200_avg	Pearson Correlation	-.107	-.290	-.478
	Sig. (2-tailed)	.297	.004	.000
	N	97	97	97
str1_150_avg	Pearson Correlation	-.006	.043	.089
	Sig. (2-tailed)	.955	.677	.381
	N	98	98	98
str2_150_avg	Pearson Correlation	.095	.008	-.084
	Sig. (2-tailed)	.351	.937	.411
	N	98	98	98
str2_250_avg	Pearson Correlation	.115	-.053	-.067
	Sig. (2-tailed)	.261	.602	.511
	N	98	98	98
TBAC6avg	Pearson Correlation	.207	.245	.330
	Sig. (2-tailed)	.041	.015	.001
	N	98	98	98
ESTpropcorr	Pearson Correlation	.287	.320	.432
	Sig. (2-tailed)	.004	.001	.000
	N	98	98	98
Memory Updating:	Pearson Correlation	.204	.383	.319
	Sig. (2-tailed)	.044	.000	.001
	N	98	98	98
Sentence Span SS Mean:	Pearson Correlation	.193	.315	.303
	Sig. (2-tailed)	.057	.002	.002
	N	98	98	98
Spatial Short Term Memory:	Pearson Correlation	.043	.193	.190
	Sig. (2-tailed)	.677	.057	.061
	N	98	98	98
AQTA1TIME	Pearson Correlation	-.073	-.314	-.298
	Sig. (2-tailed)	.479	.002	.003
	N	97	97	97
AQTA2TIME	Pearson Correlation	-.141	-.316	-.308
	Sig. (2-tailed)	.168	.002	.002
	N	98	98	98

Correlations

		TOTSEQCORR	SSQspeech	SSQmod_spatial
hm_100_avg	Pearson Correlation	-.178	-.141	-.024
	Sig. (2-tailed)	.085	.169	.814
	N	94	97	97
hm_200_avg	Pearson Correlation	-.287	-.227	-.009
	Sig. (2-tailed)	.005	.026	.930
	N	94	97	97
str1_150_avg	Pearson Correlation	.033	-.038	.085
	Sig. (2-tailed)	.753	.713	.404
	N	95	98	98
str2_150_avg	Pearson Correlation	-.045	-.278	-.075
	Sig. (2-tailed)	.666	.006	.463
	N	95	98	98
str2_250_avg	Pearson Correlation	-.057	-.269	-.077
	Sig. (2-tailed)	.584	.007	.449
	N	95	98	98
TBAC6avg	Pearson Correlation	.374	.167	.203
	Sig. (2-tailed)	.000	.101	.045
	N	95	98	98
ESTpropcorr	Pearson Correlation	.275	.233	.148
	Sig. (2-tailed)	.007	.021	.146
	N	95	98	98
Memory Updating:	Pearson Correlation	.242	.242	.322
	Sig. (2-tailed)	.018	.016	.001
	N	95	98	98
Sentence Span SS Mean:	Pearson Correlation	.415	.141	.180
	Sig. (2-tailed)	.000	.167	.077
	N	95	98	98
Spatial Short Term Memory:	Pearson Correlation	.040	.039	.172
	Sig. (2-tailed)	.699	.706	.090
	N	95	98	98
AQTA1TIME	Pearson Correlation	-.289	-.335	-.292
	Sig. (2-tailed)	.005	.001	.004
	N	94	97	97
AQTA2TIME	Pearson Correlation	-.210	-.299	-.236
	Sig. (2-tailed)	.041	.003	.019
	N	95	98	98

Correlations

		SSQmod_quality	SSQmod_overall	PTAtest	hfPTAtest
hm_100_avg	Pearson Correlation	-.144	-.128	.294	.276
	Sig. (2-tailed)	.161	.213	.003	.006
	N	96	97	97	97
hm_200_avg	Pearson Correlation	-.166	-.149	.302	.345
	Sig. (2-tailed)	.105	.145	.003	.001
	N	96	97	97	97
str1_150_avg	Pearson Correlation	.102	.060	-.090	-.180
	Sig. (2-tailed)	.319	.556	.379	.076
	N	97	98	98	98
str2_150_avg	Pearson Correlation	-.067	-.180	.201	.193
	Sig. (2-tailed)	.514	.076	.047	.057
	N	97	98	98	98
str2_250_avg	Pearson Correlation	-.065	-.180	.173	.134
	Sig. (2-tailed)	.526	.076	.088	.188
	N	97	98	98	98
TBAC6avg	Pearson Correlation	.214	.222	-.236	-.171
	Sig. (2-tailed)	.035	.028	.019	.093
	N	97	98	98	98
ESTpropcorr	Pearson Correlation	.303	.265	-.460	-.470
	Sig. (2-tailed)	.003	.008	.000	.000
	N	97	98	98	98
Memory Updating:	Pearson Correlation	.398	.366	-.207	-.148
	Sig. (2-tailed)	.000	.000	.040	.145
	N	97	98	98	98
Sentence Span SS Mean:	Pearson Correlation	.252	.218	-.180	-.147
	Sig. (2-tailed)	.013	.031	.076	.148
	N	97	98	98	98
Spatial Short Term Memory:	Pearson Correlation	.166	.142	-.247	-.197
	Sig. (2-tailed)	.103	.162	.014	.052
	N	97	98	98	98
AQTA1TIME	Pearson Correlation	-.314	-.370	.221	.302
	Sig. (2-tailed)	.002	.000	.029	.003
	N	96	97	97	97
AQTA2TIME	Pearson Correlation	-.372	-.347	.277	.341
	Sig. (2-tailed)	.000	.000	.006	.001
	N	97	98	98	98

Correlations

		AGE
hm_100_avg	Pearson Correlation	.361
	Sig. (2-tailed)	.000
	N	97
hm_200_avg	Pearson Correlation	.372
	Sig. (2-tailed)	.000
	N	97
str1_150_avg	Pearson Correlation	-.260
	Sig. (2-tailed)	.010
	N	98
str2_150_avg	Pearson Correlation	.007
	Sig. (2-tailed)	.945
	N	98
str2_250_avg	Pearson Correlation	-.033
	Sig. (2-tailed)	.744
	N	98
TBAC6avg	Pearson Correlation	-.191
	Sig. (2-tailed)	.059
	N	98
ESTpropcorr	Pearson Correlation	-.501
	Sig. (2-tailed)	.000
	N	98
Memory Updating:	Pearson Correlation	-.315
	Sig. (2-tailed)	.002
	N	98
Sentence Span SS Mean:	Pearson Correlation	-.247
	Sig. (2-tailed)	.014
	N	98
Spatial Short Term Memory:	Pearson Correlation	-.315
	Sig. (2-tailed)	.002
	N	98
AQTA1TIME	Pearson Correlation	.192
	Sig. (2-tailed)	.059
	N	97
AQTA2TIME	Pearson Correlation	.236
	Sig. (2-tailed)	.019
	N	98

Correlations

		infd_500_s_D_avg	infd_500_d_D_avg	infd_1000_s_D_avg
AQTA3TIME	Pearson Correlation	.142	.114	.098
	Sig. (2-tailed)	.166	.265	.339
	N	97	97	97
TRTavg	Pearson Correlation	-.100	-.147	-.147
	Sig. (2-tailed)	.328	.147	.150
	N	98	98	98
iSPIN_Q_Overall	Pearson Correlation	-.163	-.181	-.161
	Sig. (2-tailed)	.119	.082	.123
	N	93	93	93
Time-compressed SPIN:	Pearson Correlation	-.265	-.252	-.238
	Sig. (2-tailed)	.008	.012	.018
	N	98	98	98
ISPIN PL MEAN:	Pearson Correlation	-.218	-.134	-.250
	Sig. (2-tailed)	.031	.187	.013
	N	98	98	98
ISPIN PH MEAN:	Pearson Correlation	-.272	-.233	-.236
	Sig. (2-tailed)	.007	.021	.019
	N	98	98	98
BSPIN PL MEAN:	Pearson Correlation	-.171	-.154	-.147
	Sig. (2-tailed)	.093	.129	.148
	N	98	98	98
BSPIN PH MEAN:	Pearson Correlation	-.157	-.175	-.149
	Sig. (2-tailed)	.122	.084	.142
	N	98	98	98
No distractor:	Pearson Correlation	-.215	-.204	-.123
	Sig. (2-tailed)	.034	.044	.226
	N	98	98	98
Simultaneous:	Pearson Correlation	-.283	-.265	-.215
	Sig. (2-tailed)	.005	.008	.033
	N	98	98	98
Fo 6ST separation:	Pearson Correlation	-.402	-.320	-.325
	Sig. (2-tailed)	.000	.001	.001
	N	98	98	98
Backwards 6ST distract:	Pearson Correlation	-.294	-.245	-.165
	Sig. (2-tailed)	.003	.015	.104
	N	98	98	98

Correlations

		infd_1000_d_D_avg	mdetbb_20_avg	mdi_5_dT_avg
AQTA3TIME	Pearson Correlation	.085	.266	.131
	Sig. (2-tailed)	.409	.009	.203
	N	97	96	96
TRTavg	Pearson Correlation	-.148	-.125	-.128
	Sig. (2-tailed)	.147	.222	.210
	N	98	97	97
iSPIN_Q_Overall	Pearson Correlation	-.198	-.159	-.012
	Sig. (2-tailed)	.057	.130	.913
	N	93	92	92
Time-compressed SPIN:	Pearson Correlation	-.297	-.120	-.167
	Sig. (2-tailed)	.003	.242	.101
	N	98	97	97
ISPIN PL MEAN:	Pearson Correlation	-.246	-.173	-.135
	Sig. (2-tailed)	.014	.090	.188
	N	98	97	97
ISPIN PH MEAN:	Pearson Correlation	-.280	-.221	-.084
	Sig. (2-tailed)	.005	.030	.412
	N	98	97	97
BSPIN PL MEAN:	Pearson Correlation	-.209	-.072	-.134
	Sig. (2-tailed)	.039	.485	.190
	N	98	97	97
BSPIN PH MEAN:	Pearson Correlation	-.169	-.108	-.025
	Sig. (2-tailed)	.096	.291	.811
	N	98	97	97
No distractor:	Pearson Correlation	-.194	-.133	-.032
	Sig. (2-tailed)	.056	.193	.759
	N	98	97	97
Simultaneous:	Pearson Correlation	-.296	-.120	-.033
	Sig. (2-tailed)	.003	.243	.751
	N	98	97	97
Fo 6ST separation:	Pearson Correlation	-.344	-.247	-.164
	Sig. (2-tailed)	.001	.015	.109
	N	98	97	97
Backwards 6ST distract:	Pearson Correlation	-.257	-.116	-.058
	Sig. (2-tailed)	.010	.259	.571
	N	98	97	97

Correlations

		mdi_10_dT_avg	mdi_10_iT_avg	mdi_20_dT_avg
AQTA3TIME	Pearson Correlation	.109	-.059	.101
	Sig. (2-tailed)	.292	.570	.327
	N	96	96	96
TRTavg	Pearson Correlation	-.235	-.055	-.215
	Sig. (2-tailed)	.021	.594	.034
	N	97	97	97
iSPIN_Q_Overall	Pearson Correlation	.013	.066	-.014
	Sig. (2-tailed)	.903	.531	.892
	N	92	92	92
Time-compressed SPIN:	Pearson Correlation	-.062	.220	-.016
	Sig. (2-tailed)	.549	.031	.875
	N	97	97	97
ISPIN PL MEAN:	Pearson Correlation	-.121	.108	-.115
	Sig. (2-tailed)	.236	.293	.262
	N	97	97	97
ISPIN PH MEAN:	Pearson Correlation	-.171	.120	-.130
	Sig. (2-tailed)	.094	.242	.205
	N	97	97	97
BSPIN PL MEAN:	Pearson Correlation	-.068	.183	-.079
	Sig. (2-tailed)	.506	.072	.442
	N	97	97	97
BSPIN PH MEAN:	Pearson Correlation	-.028	.074	-.055
	Sig. (2-tailed)	.787	.473	.591
	N	97	97	97
No distractor:	Pearson Correlation	-.075	.080	-.019
	Sig. (2-tailed)	.462	.438	.852
	N	97	97	97
Simultaneous:	Pearson Correlation	.026	-.016	-.039
	Sig. (2-tailed)	.803	.880	.705
	N	97	97	97
Fo 6ST separation:	Pearson Correlation	-.220	.004	-.209
	Sig. (2-tailed)	.031	.969	.040
	N	97	97	97
Backwards 6ST distract:	Pearson Correlation	-.112	.072	-.098
	Sig. (2-tailed)	.276	.485	.339
	N	97	97	97

Correlations

		mld_250_p_avg	mld_500_p_avg	iso_FF_avg
AQTA3TIME	Pearson Correlation	.239	.354	.282
	Sig. (2-tailed)	.022	.001	.005
	N	92	92	97
TRTavg	Pearson Correlation	-.424	-.530	-.120
	Sig. (2-tailed)	.000	.000	.238
	N	93	93	98
iSPIN_Q_Overall	Pearson Correlation	-.327	-.362	-.080
	Sig. (2-tailed)	.002	.001	.447
	N	88	88	93
Time-compressed SPIN:	Pearson Correlation	-.387	-.450	-.170
	Sig. (2-tailed)	.000	.000	.094
	N	93	93	98
ISPIN PL MEAN:	Pearson Correlation	-.367	-.450	-.181
	Sig. (2-tailed)	.000	.000	.075
	N	93	93	98
ISPIN PH MEAN:	Pearson Correlation	-.381	-.391	-.212
	Sig. (2-tailed)	.000	.000	.036
	N	93	93	98
BSPIN PL MEAN:	Pearson Correlation	-.358	-.447	-.156
	Sig. (2-tailed)	.000	.000	.124
	N	93	93	98
BSPIN PH MEAN:	Pearson Correlation	-.294	-.248	-.039
	Sig. (2-tailed)	.004	.017	.704
	N	93	93	98
No distractor:	Pearson Correlation	-.232	-.201	-.062
	Sig. (2-tailed)	.025	.054	.545
	N	93	93	98
Simultaneous:	Pearson Correlation	-.170	-.149	-.178
	Sig. (2-tailed)	.103	.153	.079
	N	93	93	98
Fo 6ST separation:	Pearson Correlation	-.332	-.374	-.141
	Sig. (2-tailed)	.001	.000	.168
	N	93	93	98
Backwards 6ST distract:	Pearson Correlation	-.259	-.222	-.033
	Sig. (2-tailed)	.012	.033	.746
	N	93	93	98

Correlations

		hm_100_avg	hm_200_avg	str1_150_avg
AQTA3TIME	Pearson Correlation	.179	.186	-.061
	Sig. (2-tailed)	.081	.069	.553
	N	96	96	97
TRTavg	Pearson Correlation	-.271	-.446	.127
	Sig. (2-tailed)	.007	.000	.211
	N	97	97	98
iSPIN_Q_Overall	Pearson Correlation	-.208	-.187	.170
	Sig. (2-tailed)	.047	.075	.104
	N	92	92	93
Time-compressed SPIN:	Pearson Correlation	-.387	-.420	.080
	Sig. (2-tailed)	.000	.000	.434
	N	97	97	98
ISPIN PL MEAN:	Pearson Correlation	-.387	-.398	.083
	Sig. (2-tailed)	.000	.000	.414
	N	97	97	98
ISPIN PH MEAN:	Pearson Correlation	-.413	-.454	.137
	Sig. (2-tailed)	.000	.000	.178
	N	97	97	98
BSPIN PL MEAN:	Pearson Correlation	-.329	-.410	.142
	Sig. (2-tailed)	.001	.000	.164
	N	97	97	98
BSPIN PH MEAN:	Pearson Correlation	-.219	-.243	.209
	Sig. (2-tailed)	.031	.017	.039
	N	97	97	98
No distractor:	Pearson Correlation	-.364	-.363	.028
	Sig. (2-tailed)	.000	.000	.781
	N	97	97	98
Simultaneous:	Pearson Correlation	-.124	-.107	-.006
	Sig. (2-tailed)	.226	.297	.955
	N	97	97	98
Fo 6ST separation:	Pearson Correlation	-.398	-.290	.043
	Sig. (2-tailed)	.000	.004	.677
	N	97	97	98
Backwards 6ST distract:	Pearson Correlation	-.395	-.478	.089
	Sig. (2-tailed)	.000	.000	.381
	N	97	97	98

Correlations

		str2_150_avg	str2_250_avg	TBAC6avg
AQTA3TIME	Pearson Correlation	.115	.115	-.354
	Sig. (2-tailed)	.260	.262	.000
	N	97	97	97
TRTavg	Pearson Correlation	.039	.018	.401
	Sig. (2-tailed)	.703	.857	.000
	N	98	98	98
iSPIN_Q_Overall	Pearson Correlation	.023	.009	.495
	Sig. (2-tailed)	.826	.932	.000
	N	93	93	93
Time-compressed SPIN:	Pearson Correlation	-.208	-.211	.448
	Sig. (2-tailed)	.040	.037	.000
	N	98	98	98
ISPIN PL MEAN:	Pearson Correlation	.009	.002	.258
	Sig. (2-tailed)	.932	.984	.010
	N	98	98	98
ISPIN PH MEAN:	Pearson Correlation	-.049	-.071	.377
	Sig. (2-tailed)	.629	.487	.000
	N	98	98	98
BSPIN PL MEAN:	Pearson Correlation	-.191	-.157	.268
	Sig. (2-tailed)	.060	.122	.008
	N	98	98	98
BSPIN PH MEAN:	Pearson Correlation	-.087	-.096	.451
	Sig. (2-tailed)	.394	.348	.000
	N	98	98	98
No distractor:	Pearson Correlation	-.149	-.132	.305
	Sig. (2-tailed)	.142	.194	.002
	N	98	98	98
Simultaneous:	Pearson Correlation	.095	.115	.207
	Sig. (2-tailed)	.351	.261	.041
	N	98	98	98
Fo 6ST separation:	Pearson Correlation	.008	-.053	.245
	Sig. (2-tailed)	.937	.602	.015
	N	98	98	98
Backwards 6ST distract:	Pearson Correlation	-.084	-.067	.330
	Sig. (2-tailed)	.411	.511	.001
	N	98	98	98

Correlations

		ESTpropcorr	Memory Updating:	Sentence Span SS Mean:
AQTA3TIME	Pearson Correlation	-.292	-.471	-.481
	Sig. (2-tailed)	.004	.000	.000
	N	97	97	97
TRTavg	Pearson Correlation	.073	.394	.539
	Sig. (2-tailed)	.475	.000	.000
	N	98	98	98
iSPIN_Q_Overall	Pearson Correlation	.250	.353	.514
	Sig. (2-tailed)	.016	.001	.000
	N	93	93	93
Time-compressed SPIN:	Pearson Correlation	.485	.337	.484
	Sig. (2-tailed)	.000	.001	.000
	N	98	98	98
ISPIN PL MEAN:	Pearson Correlation	.449	.372	.380
	Sig. (2-tailed)	.000	.000	.000
	N	98	98	98
ISPIN PH MEAN:	Pearson Correlation	.537	.360	.470
	Sig. (2-tailed)	.000	.000	.000
	N	98	98	98
BSPIN PL MEAN:	Pearson Correlation	.537	.358	.430
	Sig. (2-tailed)	.000	.000	.000
	N	98	98	98
BSPIN PH MEAN:	Pearson Correlation	.317	.302	.542
	Sig. (2-tailed)	.001	.002	.000
	N	98	98	98
No distractor:	Pearson Correlation	.312	.239	.462
	Sig. (2-tailed)	.002	.018	.000
	N	98	98	98
Simultaneous:	Pearson Correlation	.287	.204	.193
	Sig. (2-tailed)	.004	.044	.057
	N	98	98	98
Fo 6ST separation:	Pearson Correlation	.320	.383	.315
	Sig. (2-tailed)	.001	.000	.002
	N	98	98	98
Backwards 6ST distract:	Pearson Correlation	.432	.319	.303
	Sig. (2-tailed)	.000	.001	.002
	N	98	98	98

Correlations

		Spatial Short Term Memory:	AQTA1TIME	AQTA2TIME
AQTA3TIME	Pearson Correlation	-.194	.716	.771
	Sig. (2-tailed)	.056	.000	.000
	N	97	97	97
TRTavg	Pearson Correlation	.216	-.422	-.358
	Sig. (2-tailed)	.033	.000	.000
	N	98	97	98
iSPIN_Q_Overall	Pearson Correlation	.145	-.144	-.443
	Sig. (2-tailed)	.165	.171	.000
	N	93	92	93
Time-compressed SPIN:	Pearson Correlation	.157	-.342	-.476
	Sig. (2-tailed)	.122	.001	.000
	N	98	97	98
ISPIN PL MEAN:	Pearson Correlation	.263	-.278	-.339
	Sig. (2-tailed)	.009	.006	.001
	N	98	97	98
ISPIN PH MEAN:	Pearson Correlation	.156	-.288	-.363
	Sig. (2-tailed)	.125	.004	.000
	N	98	97	98
BSPIN PL MEAN:	Pearson Correlation	.193	-.367	-.484
	Sig. (2-tailed)	.057	.000	.000
	N	98	97	98
BSPIN PH MEAN:	Pearson Correlation	.046	-.150	-.388
	Sig. (2-tailed)	.656	.142	.000
	N	98	97	98
No distractor:	Pearson Correlation	.166	-.164	-.246
	Sig. (2-tailed)	.102	.108	.015
	N	98	97	98
Simultaneous:	Pearson Correlation	.043	-.073	-.141
	Sig. (2-tailed)	.677	.479	.168
	N	98	97	98
Fo 6ST separation:	Pearson Correlation	.193	-.314	-.316
	Sig. (2-tailed)	.057	.002	.002
	N	98	97	98
Backwards 6ST distract:	Pearson Correlation	.190	-.298	-.308
	Sig. (2-tailed)	.061	.003	.002
	N	98	97	98

Correlations

		AQTA3TIME	TRTavg	iSPIN_Q_Overall
AQTA3TIME	Pearson Correlation	1	-.394	-.376
	Sig. (2-tailed)		.000	.000
	N	97	97	92
TRTavg	Pearson Correlation	-.394	1	.441
	Sig. (2-tailed)	.000		.000
	N	97	98	93
iSPIN_Q_Overall	Pearson Correlation	-.376	.441	1
	Sig. (2-tailed)	.000	.000	
	N	92	93	93
Time-compressed SPIN:	Pearson Correlation	-.481	.440	.713
	Sig. (2-tailed)	.000	.000	.000
	N	97	98	93
ISPIN PL MEAN:	Pearson Correlation	-.376	.430	.550
	Sig. (2-tailed)	.000	.000	.000
	N	97	98	93
ISPIN PH MEAN:	Pearson Correlation	-.431	.432	.647
	Sig. (2-tailed)	.000	.000	.000
	N	97	98	93
BSPIN PL MEAN:	Pearson Correlation	-.472	.449	.650
	Sig. (2-tailed)	.000	.000	.000
	N	97	98	93
BSPIN PH MEAN:	Pearson Correlation	-.342	.385	.833
	Sig. (2-tailed)	.001	.000	.000
	N	97	98	93
No distractor:	Pearson Correlation	-.220	.203	.473
	Sig. (2-tailed)	.030	.045	.000
	N	97	98	93
Simultaneous:	Pearson Correlation	-.141	.120	.180
	Sig. (2-tailed)	.168	.238	.084
	N	97	98	93
Fo 6ST separation:	Pearson Correlation	-.342	.331	.378
	Sig. (2-tailed)	.001	.001	.000
	N	97	98	93
Backwards 6ST distract:	Pearson Correlation	-.321	.282	.434
	Sig. (2-tailed)	.001	.005	.000
	N	97	98	93

Correlations

		Time-compressed SPIN:	ISPIN PL MEAN:	ISPIN PH MEAN:
AQTA3TIME	Pearson Correlation	-.481	-.376	-.431
	Sig. (2-tailed)	.000	.000	.000
	N	97	97	97
TRTavg	Pearson Correlation	.440	.430	.432
	Sig. (2-tailed)	.000	.000	.000
	N	98	98	98
iSPIN_Q_Overall	Pearson Correlation	.713	.550	.647
	Sig. (2-tailed)	.000	.000	.000
	N	93	93	93
Time-compressed SPIN:	Pearson Correlation	1	.642	.802
	Sig. (2-tailed)		.000	.000
	N	98	98	98
ISPIN PL MEAN:	Pearson Correlation	.642	1	.706
	Sig. (2-tailed)	.000		.000
	N	98	98	98
ISPIN PH MEAN:	Pearson Correlation	.802	.706	1
	Sig. (2-tailed)	.000	.000	
	N	98	98	98
BSPIN PL MEAN:	Pearson Correlation	.823	.756	.766
	Sig. (2-tailed)	.000	.000	.000
	N	98	98	98
BSPIN PH MEAN:	Pearson Correlation	.728	.457	.730
	Sig. (2-tailed)	.000	.000	.000
	N	98	98	98
No distractor:	Pearson Correlation	.445	.312	.478
	Sig. (2-tailed)	.000	.002	.000
	N	98	98	98
Simultaneous:	Pearson Correlation	.164	.168	.254
	Sig. (2-tailed)	.107	.098	.011
	N	98	98	98
Fo 6ST separation:	Pearson Correlation	.486	.470	.505
	Sig. (2-tailed)	.000	.000	.000
	N	98	98	98
Backwards 6ST distract:	Pearson Correlation	.588	.454	.629
	Sig. (2-tailed)	.000	.000	.000
	N	98	98	98

Correlations

		BSPIN PL MEAN:	BSPIN PH MEAN:	No distractor:
AQTA3TIME	Pearson Correlation	-.472	-.342	-.220
	Sig. (2-tailed)	.000	.001	.030
	N	97	97	97
TRTavg	Pearson Correlation	.449	.385	.203
	Sig. (2-tailed)	.000	.000	.045
	N	98	98	98
iSPIN_Q_Overall	Pearson Correlation	.650	.833	.473
	Sig. (2-tailed)	.000	.000	.000
	N	93	93	93
Time-compressed SPIN:	Pearson Correlation	.823	.728	.445
	Sig. (2-tailed)	.000	.000	.000
	N	98	98	98
ISPIN PL MEAN:	Pearson Correlation	.756	.457	.312
	Sig. (2-tailed)	.000	.000	.002
	N	98	98	98
ISPIN PH MEAN:	Pearson Correlation	.766	.730	.478
	Sig. (2-tailed)	.000	.000	.000
	N	98	98	98
BSPIN PL MEAN:	Pearson Correlation	1	.626	.367
	Sig. (2-tailed)		.000	.000
	N	98	98	98
BSPIN PH MEAN:	Pearson Correlation	.626	1	.453
	Sig. (2-tailed)	.000		.000
	N	98	98	98
No distractor:	Pearson Correlation	.367	.453	1
	Sig. (2-tailed)	.000	.000	
	N	98	98	98
Simultaneous:	Pearson Correlation	.121	.167	.229
	Sig. (2-tailed)	.236	.101	.023
	N	98	98	98
Fo 6ST separation:	Pearson Correlation	.435	.320	.304
	Sig. (2-tailed)	.000	.001	.002
	N	98	98	98
Backwards 6ST distract:	Pearson Correlation	.524	.580	.374
	Sig. (2-tailed)	.000	.000	.000
	N	98	98	98

Correlations

		Simultaneous:	Fo 6ST separation:	Backwards 6ST distract:
AQTA3TIME	Pearson Correlation	-.141	-.342	-.321
	Sig. (2-tailed)	.168	.001	.001
	N	97	97	97
TRTavg	Pearson Correlation	.120	.331	.282
	Sig. (2-tailed)	.238	.001	.005
	N	98	98	98
iSPIN_Q_Overall	Pearson Correlation	.180	.378	.434
	Sig. (2-tailed)	.084	.000	.000
	N	93	93	93
Time-compressed SPIN:	Pearson Correlation	.164	.486	.588
	Sig. (2-tailed)	.107	.000	.000
	N	98	98	98
ISPIN PL MEAN:	Pearson Correlation	.168	.470	.454
	Sig. (2-tailed)	.098	.000	.000
	N	98	98	98
ISPIN PH MEAN:	Pearson Correlation	.254	.505	.629
	Sig. (2-tailed)	.011	.000	.000
	N	98	98	98
BSPIN PL MEAN:	Pearson Correlation	.121	.435	.524
	Sig. (2-tailed)	.236	.000	.000
	N	98	98	98
BSPIN PH MEAN:	Pearson Correlation	.167	.320	.580
	Sig. (2-tailed)	.101	.001	.000
	N	98	98	98
No distractor:	Pearson Correlation	.229	.304	.374
	Sig. (2-tailed)	.023	.002	.000
	N	98	98	98
Simultaneous:	Pearson Correlation	1	.354	.250
	Sig. (2-tailed)		.000	.013
	N	98	98	98
Fo 6ST separation:	Pearson Correlation	.354	1	.467
	Sig. (2-tailed)	.000		.000
	N	98	98	98
Backwards 6ST distract:	Pearson Correlation	.250	.467	1
	Sig. (2-tailed)	.013	.000	
	N	98	98	98

Correlations

		TOTSEQCOR R	SSQspeech	SSQmod_spa tial
AQTA3TIME	Pearson Correlation	-.231	-.176	-.228
	Sig. (2-tailed)	.025	.084	.025
	N	94	97	97
TRTavg	Pearson Correlation	.307	.152	.195
	Sig. (2-tailed)	.002	.134	.054
	N	95	98	98
iSPIN_Q_Overall	Pearson Correlation	.272	.187	.119
	Sig. (2-tailed)	.010	.073	.256
	N	90	93	93
Time-compressed SPIN:	Pearson Correlation	.381	.334	.180
	Sig. (2-tailed)	.000	.001	.077
	N	95	98	98
ISPIN PL MEAN:	Pearson Correlation	.446	.163	.115
	Sig. (2-tailed)	.000	.108	.261
	N	95	98	98
ISPIN PH MEAN:	Pearson Correlation	.426	.247	.132
	Sig. (2-tailed)	.000	.014	.195
	N	95	98	98
BSPIN PL MEAN:	Pearson Correlation	.369	.319	.216
	Sig. (2-tailed)	.000	.001	.033
	N	95	98	98
BSPIN PH MEAN:	Pearson Correlation	.331	.202	.109
	Sig. (2-tailed)	.001	.046	.285
	N	95	98	98
No distractor:	Pearson Correlation	.165	.193	-.096
	Sig. (2-tailed)	.110	.057	.349
	N	95	98	98
Simultaneous:	Pearson Correlation	.282	-.027	-.021
	Sig. (2-tailed)	.006	.794	.840
	N	95	98	98
Fo 6ST separation:	Pearson Correlation	.458	.126	.081
	Sig. (2-tailed)	.000	.216	.430
	N	95	98	98
Backwards 6ST distract:	Pearson Correlation	.400	.299	.109
	Sig. (2-tailed)	.000	.003	.285
	N	95	98	98

Correlations

		SSQmod_quality	SSQmod_overall	PTAtest	hfPTAtest
AQTA3TIME	Pearson Correlation	-.319	-.283	.289	.362
	Sig. (2-tailed)	.002	.005	.004	.000
	N	96	97	97	97
TRTavg	Pearson Correlation	.236	.229	-.239	-.246
	Sig. (2-tailed)	.020	.023	.018	.014
	N	97	98	98	98
iSPIN_Q_Overall	Pearson Correlation	.223	.212	-.461	-.399
	Sig. (2-tailed)	.033	.042	.000	.000
	N	92	93	93	93
Time-compressed SPIN:	Pearson Correlation	.363	.345	-.646	-.661
	Sig. (2-tailed)	.000	.000	.000	.000
	N	97	98	98	98
ISPIN PL MEAN:	Pearson Correlation	.298	.224	-.409	-.402
	Sig. (2-tailed)	.003	.027	.000	.000
	N	97	98	98	98
ISPIN PH MEAN:	Pearson Correlation	.272	.262	-.499	-.487
	Sig. (2-tailed)	.007	.009	.000	.000
	N	97	98	98	98
BSPIN PL MEAN:	Pearson Correlation	.413	.369	-.581	-.657
	Sig. (2-tailed)	.000	.000	.000	.000
	N	97	98	98	98
BSPIN PH MEAN:	Pearson Correlation	.185	.206	-.373	-.388
	Sig. (2-tailed)	.070	.042	.000	.000
	N	97	98	98	98
No distractor:	Pearson Correlation	.109	.077	-.117	-.115
	Sig. (2-tailed)	.287	.451	.251	.258
	N	97	98	98	98
Simultaneous:	Pearson Correlation	-.027	-.030	-.075	-.040
	Sig. (2-tailed)	.790	.768	.463	.698
	N	97	98	98	98
Fo 6ST separation:	Pearson Correlation	.082	.124	-.313	-.304
	Sig. (2-tailed)	.424	.222	.002	.002
	N	97	98	98	98
Backwards 6ST distract:	Pearson Correlation	.169	.234	-.378	-.487
	Sig. (2-tailed)	.099	.020	.000	.000
	N	97	98	98	98

Correlations

		AGE
AQTA3TIME	Pearson Correlation	.338
	Sig. (2-tailed)	.001
	N	97
TRTavg	Pearson Correlation	-.239
	Sig. (2-tailed)	.018
	N	98
iSPIN_Q_Overall	Pearson Correlation	-.367
	Sig. (2-tailed)	.000
	N	93
Time-compressed SPIN:	Pearson Correlation	-.498
	Sig. (2-tailed)	.000
	N	98
ISPIN PL MEAN:	Pearson Correlation	-.444
	Sig. (2-tailed)	.000
	N	98
ISPIN PH MEAN:	Pearson Correlation	-.472
	Sig. (2-tailed)	.000
	N	98
BSPIN PL MEAN:	Pearson Correlation	-.524
	Sig. (2-tailed)	.000
	N	98
BSPIN PH MEAN:	Pearson Correlation	-.339
	Sig. (2-tailed)	.001
	N	98
No distractor:	Pearson Correlation	-.264
	Sig. (2-tailed)	.009
	N	98
Simultaneous:	Pearson Correlation	-.151
	Sig. (2-tailed)	.137
	N	98
Fo 6ST separation:	Pearson Correlation	-.381
	Sig. (2-tailed)	.000
	N	98
Backwards 6ST distract:	Pearson Correlation	-.543
	Sig. (2-tailed)	.000
	N	98

Correlations

		infd_500_s_D_avg	infd_500_d_D_avg	infd_1000_s_D_avg
TOTSEQCORR	Pearson Correlation	-.380	-.193	-.403
	Sig. (2-tailed)	.000	.061	.000
	N	95	95	95
SSQspeech	Pearson Correlation	-.082	-.011	.049
	Sig. (2-tailed)	.420	.918	.633
	N	98	98	98
SSQmod_spatial	Pearson Correlation	.040	.057	.016
	Sig. (2-tailed)	.699	.580	.873
	N	98	98	98
SSQmod_quality	Pearson Correlation	-.044	.020	-.066
	Sig. (2-tailed)	.667	.847	.519
	N	97	97	97
SSQmod_overall	Pearson Correlation	-.033	.026	.006
	Sig. (2-tailed)	.744	.803	.953
	N	98	98	98
PTAtest	Pearson Correlation	.088	.094	.058
	Sig. (2-tailed)	.387	.358	.573
	N	98	98	98
hfPTAtest	Pearson Correlation	.042	.063	-.055
	Sig. (2-tailed)	.683	.539	.588
	N	98	98	98
AGE	Pearson Correlation	.114	.133	.063
	Sig. (2-tailed)	.262	.190	.539
	N	98	98	98

Correlations

		infd_1000_d_D_avg	mdetbb_20_avg	mdi_5_dT_avg
TOTSEQCORR	Pearson Correlation	-.294	-.256	-.227
	Sig. (2-tailed)	.004	.013	.028
	N	95	94	94
SSQspeech	Pearson Correlation	.030	-.077	-.160
	Sig. (2-tailed)	.772	.451	.117
	N	98	97	97
SSQmod_spatial	Pearson Correlation	.041	-.206	-.218
	Sig. (2-tailed)	.690	.043	.032
	N	98	97	97
SSQmod_quality	Pearson Correlation	-.008	-.172	-.283
	Sig. (2-tailed)	.938	.094	.005
	N	97	96	96
SSQmod_overall	Pearson Correlation	.028	-.179	-.247
	Sig. (2-tailed)	.786	.079	.015
	N	98	97	97
PTAtest	Pearson Correlation	.091	.014	.097
	Sig. (2-tailed)	.375	.890	.345
	N	98	97	97
hfPTAtest	Pearson Correlation	.022	-.108	.090
	Sig. (2-tailed)	.833	.291	.378
	N	98	97	97
AGE	Pearson Correlation	.195	.008	-.070
	Sig. (2-tailed)	.055	.940	.495
	N	98	97	97

Correlations

		mdi_10_dT_avg	mdi_10_iT_avg	mdi_20_dT_avg
TOTSEQCORR	Pearson Correlation	-.185	.053	-.220
	Sig. (2-tailed)	.074	.609	.033
	N	94	94	94
SSQspeech	Pearson Correlation	-.160	.234	-.014
	Sig. (2-tailed)	.116	.021	.893
	N	97	97	97
SSQmod_spatial	Pearson Correlation	-.197	-.015	-.191
	Sig. (2-tailed)	.053	.883	.060
	N	97	97	97
SSQmod_quality	Pearson Correlation	-.204	.080	-.165
	Sig. (2-tailed)	.046	.440	.109
	N	96	96	96
SSQmod_overall	Pearson Correlation	-.214	.111	-.136
	Sig. (2-tailed)	.035	.278	.184
	N	97	97	97
PTAtest	Pearson Correlation	-.005	-.248	-.048
	Sig. (2-tailed)	.960	.014	.638
	N	97	97	97
hfPTAtest	Pearson Correlation	-.044	-.240	-.052
	Sig. (2-tailed)	.669	.018	.610
	N	97	97	97
AGE	Pearson Correlation	-.099	-.160	-.083
	Sig. (2-tailed)	.336	.117	.420
	N	97	97	97

Correlations

		mld_250_p_avg	mld_500_p_avg	iso_FF_avg
TOTSEQCORR	Pearson Correlation	-.294	-.290	-.237
	Sig. (2-tailed)	.005	.005	.021
	N	91	91	95
SSQspeech	Pearson Correlation	-.081	-.120	-.098
	Sig. (2-tailed)	.440	.253	.338
	N	93	93	98
SSQmod_spatial	Pearson Correlation	-.217	-.203	-.202
	Sig. (2-tailed)	.036	.051	.046
	N	93	93	98
SSQmod_quality	Pearson Correlation	-.270	-.263	-.283
	Sig. (2-tailed)	.009	.011	.005
	N	92	92	97
SSQmod_overall	Pearson Correlation	-.226	-.236	-.213
	Sig. (2-tailed)	.029	.023	.035
	N	93	93	98
PTAtest	Pearson Correlation	.471	.450	.190
	Sig. (2-tailed)	.000	.000	.061
	N	93	93	98
hfPTAtest	Pearson Correlation	.378	.422	.062
	Sig. (2-tailed)	.000	.000	.544
	N	93	93	98
AGE	Pearson Correlation	.275	.349	-.004
	Sig. (2-tailed)	.008	.001	.971
	N	93	93	98

Correlations

		hm_100_avg	hm_200_avg	str1_150_avg
TOTSEQCORR	Pearson Correlation	-.178	-.287	.033
	Sig. (2-tailed)	.085	.005	.753
	N	94	94	95
SSQspeech	Pearson Correlation	-.141	-.227	-.038
	Sig. (2-tailed)	.169	.026	.713
	N	97	97	98
SSQmod_spatial	Pearson Correlation	-.024	-.009	.085
	Sig. (2-tailed)	.814	.930	.404
	N	97	97	98
SSQmod_quality	Pearson Correlation	-.144	-.166	.102
	Sig. (2-tailed)	.161	.105	.319
	N	96	96	97
SSQmod_overall	Pearson Correlation	-.128	-.149	.060
	Sig. (2-tailed)	.213	.145	.556
	N	97	97	98
PTAtest	Pearson Correlation	.294	.302	-.090
	Sig. (2-tailed)	.003	.003	.379
	N	97	97	98
hfPTAtest	Pearson Correlation	.276	.345	-.180
	Sig. (2-tailed)	.006	.001	.076
	N	97	97	98
AGE	Pearson Correlation	.361	.372	-.260
	Sig. (2-tailed)	.000	.000	.010
	N	97	97	98

Correlations

		str2_150_avg	str2_250_avg	TBAC6avg
TOTSEQCORR	Pearson Correlation	-.045	-.057	.374
	Sig. (2-tailed)	.666	.584	.000
	N	95	95	95
SSQspeech	Pearson Correlation	-.278	-.269	.167
	Sig. (2-tailed)	.006	.007	.101
	N	98	98	98
SSQmod_spatial	Pearson Correlation	-.075	-.077	.203
	Sig. (2-tailed)	.463	.449	.045
	N	98	98	98
SSQmod_quality	Pearson Correlation	-.067	-.065	.214
	Sig. (2-tailed)	.514	.526	.035
	N	97	97	97
SSQmod_overall	Pearson Correlation	-.180	-.180	.222
	Sig. (2-tailed)	.076	.076	.028
	N	98	98	98
PTAtest	Pearson Correlation	.201	.173	-.236
	Sig. (2-tailed)	.047	.088	.019
	N	98	98	98
hfPTAtest	Pearson Correlation	.193	.134	-.171
	Sig. (2-tailed)	.057	.188	.093
	N	98	98	98
AGE	Pearson Correlation	.007	-.033	-.191
	Sig. (2-tailed)	.945	.744	.059
	N	98	98	98

Correlations

		ESTpropcorr	Memory Updating:	Sentence Span SS Mean:
TOTSEQCORR	Pearson Correlation	.275	.242	.415
	Sig. (2-tailed)	.007	.018	.000
	N	95	95	95
SSQspeech	Pearson Correlation	.233	.242	.141
	Sig. (2-tailed)	.021	.016	.167
	N	98	98	98
SSQmod_spatial	Pearson Correlation	.148	.322	.180
	Sig. (2-tailed)	.146	.001	.077
	N	98	98	98
SSQmod_quality	Pearson Correlation	.303	.398	.252
	Sig. (2-tailed)	.003	.000	.013
	N	97	97	97
SSQmod_overall	Pearson Correlation	.265	.366	.218
	Sig. (2-tailed)	.008	.000	.031
	N	98	98	98
PTAtest	Pearson Correlation	-.460	-.207	-.180
	Sig. (2-tailed)	.000	.040	.076
	N	98	98	98
hfPTAtest	Pearson Correlation	-.470	-.148	-.147
	Sig. (2-tailed)	.000	.145	.148
	N	98	98	98
AGE	Pearson Correlation	-.501	-.315	-.247
	Sig. (2-tailed)	.000	.002	.014
	N	98	98	98

Correlations

		Spatial Short Term Memory:	AQTA1TIME	AQTA2TIME
TOTSEQCORR	Pearson Correlation	.040	-.289	-.210
	Sig. (2-tailed)	.699	.005	.041
	N	95	94	95
SSQspeech	Pearson Correlation	.039	-.335	-.299
	Sig. (2-tailed)	.706	.001	.003
	N	98	97	98
SSQmod_spatial	Pearson Correlation	.172	-.292	-.236
	Sig. (2-tailed)	.090	.004	.019
	N	98	97	98
SSQmod_quality	Pearson Correlation	.166	-.314	-.372
	Sig. (2-tailed)	.103	.002	.000
	N	97	96	97
SSQmod_overall	Pearson Correlation	.142	-.370	-.347
	Sig. (2-tailed)	.162	.000	.000
	N	98	97	98
PTAtest	Pearson Correlation	-.247	.221	.277
	Sig. (2-tailed)	.014	.029	.006
	N	98	97	98
hfPTAtest	Pearson Correlation	-.197	.302	.341
	Sig. (2-tailed)	.052	.003	.001
	N	98	97	98
AGE	Pearson Correlation	-.315	.192	.236
	Sig. (2-tailed)	.002	.059	.019
	N	98	97	98

Correlations

		AQTA3TIME	TRTavg	iSPIN_Q_Overall
TOTSEQCORR	Pearson Correlation	-.231	.307	.272
	Sig. (2-tailed)	.025	.002	.010
	N	94	95	90
SSQspeech	Pearson Correlation	-.176	.152	.187
	Sig. (2-tailed)	.084	.134	.073
	N	97	98	93
SSQmod_spatial	Pearson Correlation	-.228	.195	.119
	Sig. (2-tailed)	.025	.054	.256
	N	97	98	93
SSQmod_quality	Pearson Correlation	-.319	.236	.223
	Sig. (2-tailed)	.002	.020	.033
	N	96	97	92
SSQmod_overall	Pearson Correlation	-.283	.229	.212
	Sig. (2-tailed)	.005	.023	.042
	N	97	98	93
PTAtest	Pearson Correlation	.289	-.239	-.461
	Sig. (2-tailed)	.004	.018	.000
	N	97	98	93
hfPTAtest	Pearson Correlation	.362	-.246	-.399
	Sig. (2-tailed)	.000	.014	.000
	N	97	98	93
AGE	Pearson Correlation	.338	-.239	-.367
	Sig. (2-tailed)	.001	.018	.000
	N	97	98	93

Correlations

		Time-compressed SPIN:	ISPIN PL MEAN:	ISPIN PH MEAN:
TOTSEQCORR	Pearson Correlation	.381	.446	.426
	Sig. (2-tailed)	.000	.000	.000
	N	95	95	95
SSQspeech	Pearson Correlation	.334	.163	.247
	Sig. (2-tailed)	.001	.108	.014
	N	98	98	98
SSQmod_spatial	Pearson Correlation	.180	.115	.132
	Sig. (2-tailed)	.077	.261	.195
	N	98	98	98
SSQmod_quality	Pearson Correlation	.363	.298	.272
	Sig. (2-tailed)	.000	.003	.007
	N	97	97	97
SSQmod_overall	Pearson Correlation	.345	.224	.262
	Sig. (2-tailed)	.000	.027	.009
	N	98	98	98
PTAtest	Pearson Correlation	-.646	-.409	-.499
	Sig. (2-tailed)	.000	.000	.000
	N	98	98	98
hfPTAtest	Pearson Correlation	-.661	-.402	-.487
	Sig. (2-tailed)	.000	.000	.000
	N	98	98	98
AGE	Pearson Correlation	-.498	-.444	-.472
	Sig. (2-tailed)	.000	.000	.000
	N	98	98	98

Correlations

		BSPIN PL MEAN:	BSPIN PH MEAN:	No distractor:
TOTSEQCORR	Pearson Correlation	.369	.331	.165
	Sig. (2-tailed)	.000	.001	.110
	N	95	95	95
SSQspeech	Pearson Correlation	.319	.202	.193
	Sig. (2-tailed)	.001	.046	.057
	N	98	98	98
SSQmod_spatial	Pearson Correlation	.216	.109	-.096
	Sig. (2-tailed)	.033	.285	.349
	N	98	98	98
SSQmod_quality	Pearson Correlation	.413	.185	.109
	Sig. (2-tailed)	.000	.070	.287
	N	97	97	97
SSQmod_overall	Pearson Correlation	.369	.206	.077
	Sig. (2-tailed)	.000	.042	.451
	N	98	98	98
PTAtest	Pearson Correlation	-.581	-.373	-.117
	Sig. (2-tailed)	.000	.000	.251
	N	98	98	98
hfPTAtest	Pearson Correlation	-.657	-.388	-.115
	Sig. (2-tailed)	.000	.000	.258
	N	98	98	98
AGE	Pearson Correlation	-.524	-.339	-.264
	Sig. (2-tailed)	.000	.001	.009
	N	98	98	98

Correlations

		Simultaneous:	Fo 6ST separation:	Backwards 6ST distract:
TOTSEQCORR	Pearson Correlation	.282	.458	.400
	Sig. (2-tailed)	.006	.000	.000
	N	95	95	95
SSQspeech	Pearson Correlation	-.027	.126	.299
	Sig. (2-tailed)	.794	.216	.003
	N	98	98	98
SSQmod_spatial	Pearson Correlation	-.021	.081	.109
	Sig. (2-tailed)	.840	.430	.285
	N	98	98	98
SSQmod_quality	Pearson Correlation	-.027	.082	.169
	Sig. (2-tailed)	.790	.424	.099
	N	97	97	97
SSQmod_overall	Pearson Correlation	-.030	.124	.234
	Sig. (2-tailed)	.768	.222	.020
	N	98	98	98
PTAtest	Pearson Correlation	-.075	-.313	-.378
	Sig. (2-tailed)	.463	.002	.000
	N	98	98	98
hfPTAtest	Pearson Correlation	-.040	-.304	-.487
	Sig. (2-tailed)	.698	.002	.000
	N	98	98	98
AGE	Pearson Correlation	-.151	-.381	-.543
	Sig. (2-tailed)	.137	.000	.000
	N	98	98	98

Correlations

		TOTSEQCORR	SSQspeech	SSQmod_spatial
TOTSEQCORR	Pearson Correlation	1	.195	.187
	Sig. (2-tailed)		.059	.069
	N	95	95	95
SSQspeech	Pearson Correlation	.195	1	.474
	Sig. (2-tailed)	.059		.000
	N	95	98	98
SSQmod_spatial	Pearson Correlation	.187	.474	1
	Sig. (2-tailed)	.069	.000	
	N	95	98	98
SSQmod_quality	Pearson Correlation	.251	.656	.644
	Sig. (2-tailed)	.014	.000	.000
	N	94	97	97
SSQmod_overall	Pearson Correlation	.246	.833	.845
	Sig. (2-tailed)	.016	.000	.000
	N	95	98	98
PTAtest	Pearson Correlation	-.046	-.366	-.274
	Sig. (2-tailed)	.658	.000	.006
	N	95	98	98
hfPTAtest	Pearson Correlation	-.056	-.353	-.246
	Sig. (2-tailed)	.590	.000	.015
	N	95	98	98
AGE	Pearson Correlation	-.195	-.072	-.052
	Sig. (2-tailed)	.058	.480	.609
	N	95	98	98

Correlations

		SSQmod_quality	SSQmod_overall	PTAtest	hfPTAtest
TOTSEQCORR	Pearson Correlation	.251	.246	-.046	-.056
	Sig. (2-tailed)	.014	.016	.658	.590
	N	94	95	95	95
SSQspeech	Pearson Correlation	.656	.833	-.366	-.353
	Sig. (2-tailed)	.000	.000	.000	.000
	N	97	98	98	98
SSQmod_spatial	Pearson Correlation	.644	.845	-.274	-.246
	Sig. (2-tailed)	.000	.000	.006	.015
	N	97	98	98	98
SSQmod_quality	Pearson Correlation	1	.878	-.390	-.370
	Sig. (2-tailed)		.000	.000	.000
	N	97	97	97	97
SSQmod_overall	Pearson Correlation	.878	1	-.404	-.379
	Sig. (2-tailed)	.000		.000	.000
	N	97	98	98	98
PTAtest	Pearson Correlation	-.390	-.404	1	.904
	Sig. (2-tailed)	.000	.000		.000
	N	97	98	98	98
hfPTAtest	Pearson Correlation	-.370	-.379	.904	1
	Sig. (2-tailed)	.000	.000	.000	
	N	97	98	98	98
AGE	Pearson Correlation	-.095	-.094	.430	.542
	Sig. (2-tailed)	.354	.356	.000	.000
	N	97	98	98	98

Correlations

		AGE
TOTSEQCORR	Pearson Correlation	-.195
	Sig. (2-tailed)	.058
	N	95
SSQspeech	Pearson Correlation	-.072
	Sig. (2-tailed)	.480
	N	98
SSQmod_spatial	Pearson Correlation	-.052
	Sig. (2-tailed)	.609
	N	98
SSQmod_quality	Pearson Correlation	-.095
	Sig. (2-tailed)	.354
	N	97
SSQmod_overall	Pearson Correlation	-.094
	Sig. (2-tailed)	.356
	N	98
PTAtest	Pearson Correlation	.430
	Sig. (2-tailed)	.000
	N	98
hfPTAtest	Pearson Correlation	.542
	Sig. (2-tailed)	.000
	N	98
AGE	Pearson Correlation	1
	Sig. (2-tailed)	
	N	98
